# Determinants of affinity, specificity, and phase separation in a supramodule from Post-synaptic density protein 95

**DOI:** 10.1016/j.isci.2022.105069

**Published:** 2022-09-05

**Authors:** Louise Laursen, Raviteja Inturi, Søren Østergaard, Per Jemth

**Affiliations:** 1 Department of Medical Biochemistry and Microbiology, Uppsala University, BMC Box 582, 75123 Uppsala, Sweden; 2 Global Research Technology, Novo Nordisk A/S, Novo Nordisk Research Park, 2760 Maalov, Denmark

**Keywords:** Protein, Protein structure aspects, Biophysics

## Abstract

The post-synaptic density (PSD) is a phase-separated membraneless compartment of proteins including PSD-95 that undergoes morphological alteration in response to synaptic activity. Here, we investigated the interactome of a three-domain supramodule, PDZ3-SH3-GK (PSG) from PSD-95 using bioinformatics to identify potential binding partners, and biophysical methods to characterize the interaction with peptides from these proteins. PSG and the single PDZ3 domain bound similar peptides, but with different specificity. Furthermore, we found that the protein ADGRB1 formed liquid droplets with the PSG supramodule, extending the model for PSD formation. Moreover, certain mutations, introduced outside of the binding pocket in PDZ3, increased the affinity and specificity of the interaction and the size of liquid droplets. Other mutations within the ligand binding pocket lead to a new binding motif specificity. Our results show how the context in terms of supertertiary structure modulates affinity, specificity, and phase separation, and how these properties can evolve by point mutation.

## Highlights

• Identification of potential binding partners for PSD-95 in the post-synaptic density
• ADGRB1 and PSD-95 undergo liquid-liquid phase separation (LLPS)
• Single domain PDZ3 cannot induce LLPS and binds weakly to ADGRB1 and SynGap
• Supertertiary structure alters the affinity, specificity, and phase separation

## Graphical abstract

**Figure:**
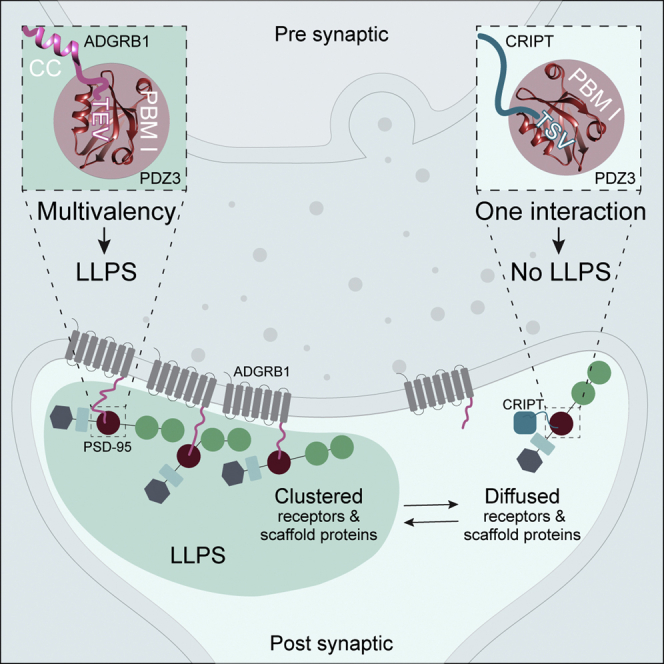

## Introduction

PSD is a membraneless compartment with a high density of proteins located between the postsynaptic plasma membrane and the cytoplasm of dendritic spines. Dendritic spines of excitatory synapses are plastic, dynamic, and change morphology in response to synaptic strength (Nishiyama and Yasuda, 2015), which is essential for synaptic plasticity, learning, and memory (Lamprecht and LeDoux, 2004). The PSD contains hundreds of different proteins with varying levels of abundance that form a disc-shaped inter-connected network (Cohen et al., 1977). Proteins are added and removed in response to synaptic strength, thus constantly remodeling the PSD, but the overall structure of the PSD is retained by a matrix of nearly immobile scaffold proteins (Blanpied et al., 2008).

**Figure 1:**
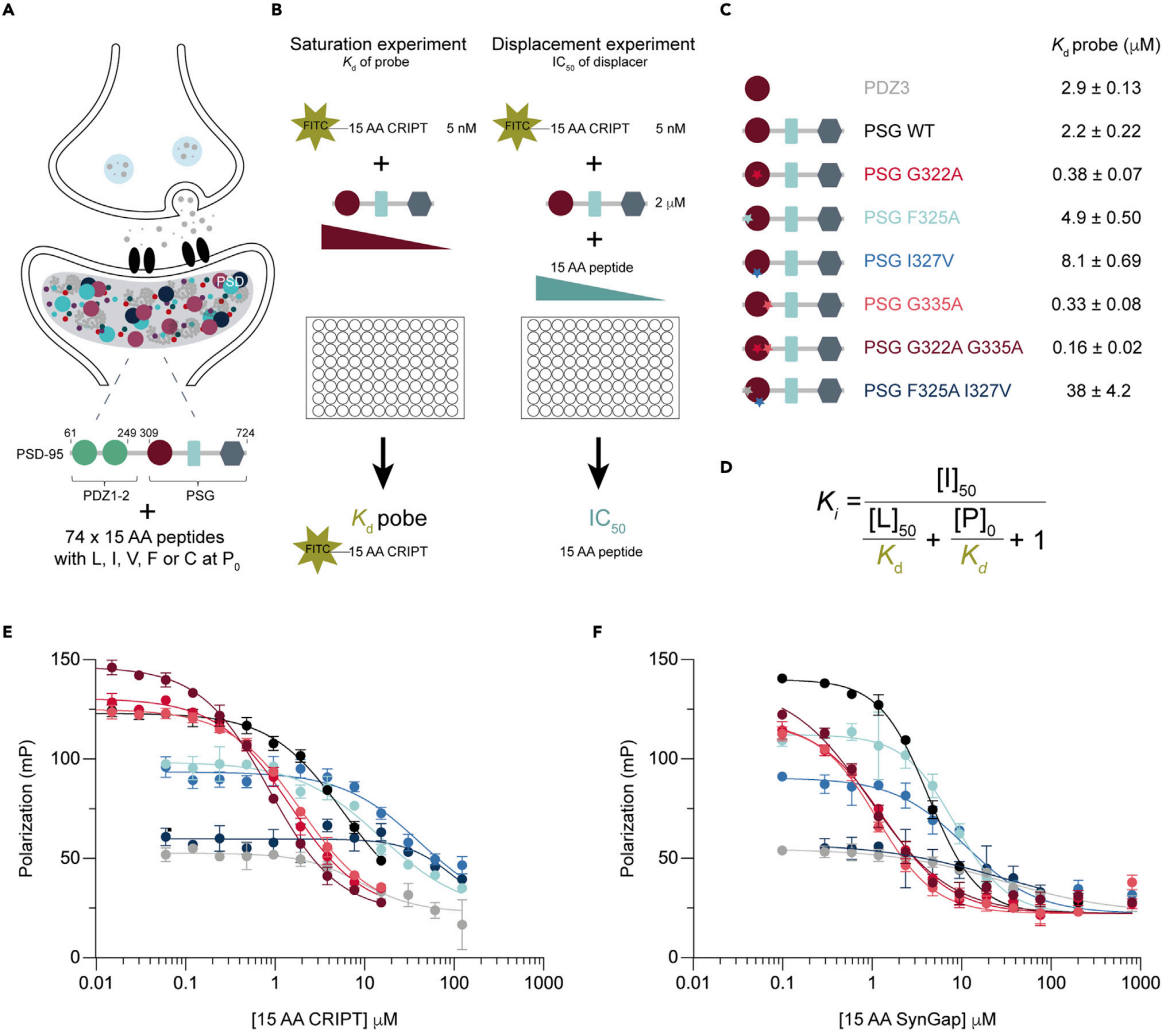
Screening of the post-synaptic density for novel PSG:peptide interactions (A) Illustration of a neuron where post-synaptic density is highlighted to visualize the crowded surroundings of PSD-95. All proteins in the PSD with Leu, Ile, Val, or Cys at their C-terminal position P_0_ were selected for screening of the interactome of PSG from PSD-95. (B) The interactome was screened by a fluorescence polarization assay. Saturation experiments were performed for PDZ3, PSG, PSG_G322A_, PSG_F325A_, PSG_I327V_, PSG_G335A_, PSG_G322A G335A_, and PSG_F325A__I327V_ to determine *K*_d_ of an FITC-labeled CRIPT-based peptide probe. Displacement experiments were performed by pre-incubating PSG or PDZ3 with the FITC-labeled probe, which was then displaced from PSG or PDZ3 by adding increasing concentration of each of the 74 peptides in separate experiments. PSG is drawn schematically with three symbols representing the PDZ (circle), SH3 (rectangle), and GK (diamond) domains. (C) Schematic illustration of the eight proteins used in the present work. Stars illustrate the number of mutations in the protein. Affinity of the FITC labeled probe was determined by saturation experiments as explained in (B). (D) Equation used to convert the obtained IC_50_ from the displacement experiment into a *K*_*i*_ value (which theoretically is equal to the equilibrium dissociation constant) for each protein:peptide interaction. [I]_50_ represents the concentration of free inhibitor (peptide) at 50% inhibition, [L]_50_ is the concentration of free FITC-15 AA CRIPT probe at 50% inhibition, [P]_0_ is the concentration of free protein at zero% inhibition, and *K*_*d*_ is the dissociation constant of protein:FITC-15 AA CRIPT complex (obtained from the separate saturation experiment) (Nikolovska-Coleska et al., 2004). (E) Fluorescence polarization-based measurement of the protein:15 AA CRIPT interaction, where the unlabeled 15 AA CRIPT displaces FITC-15 AA CRIPT. (F) Fluorescence polarization-based measurement of protein:15 AA SynGap interaction. All displacement experiments were performed in technical triplicates. The color code for each protein is shown in panel C and is used throughout the manuscript: gray (PDZ3), black (PSG WT), red (PSG_G322A_), light blue (PSG_F325A_), blue (PSG_I327V_), pink-orange (PSG_G335A_), bordeaux (PSG_G322A G335A_), and dark blue (PSG_F325A I327V_). Errors bars represent the 95% confidence intervals from three technical replicates.

PSD-95 is the most abundant scaffold protein in the PSD (Cheng et al., 2006) and is required to sustain the molecular organization of the compartment (Chen et al., 2011). PSD-95 has several binding partners in the PSD: CRIPT (Niethammer et al., 1998), SynGap (Zeng et al., 2016), NMDA receptors (Niethammer et al., 1996), cell adhesion proteins (Irie et al., 1997), and other scaffold proteins such as GKAP (Zeng et al., 2018), emphasizing its importance in a structural organization. PSD-95 is one of four paralogs in humans together with PSD-93, SAP102, and SAP97. They are members of the membrane-associated guanylate kinase family that all share three types of domains: PSD-95, Discs-large, Zona occludens 1 (PDZ), Src homology 3 (SH3), and guanylate kinase (GK) domains, respectively (Figure 1A). The supertertiary structure formed by the three domains (PDZ, SH3, and GK) is denoted the PSG supramodule. Its function depends on the interplay between the three domains and is not recapitulated by the properties of the isolated protein domains (Feng and Zhang, 2009; Laursen et al., 2020b; Rademacher et al., 2019).

The PSG forms liquid droplets in a complex with SynGap, which binds via its C-terminal to the PDZ3 domain of PSG (Zeng et al., 2016). Based on this observation Zhang and co-workers suggested that liquid-liquid phase separation (LLPS) underlies the formation of the PSD (Zeng et al., 2016). To prove that the PSD forms by LLPS, it was reconstituted by using four scaffold proteins from PSD: PSD-95, GKAP, Shank, and Homer (Zeng et al., 2018). Indeed, the reconstituted PSD-like assemblies recapitulated many properties of PSD: they could cluster receptors, selectively concentrate enzymes, promote actin bundle formation and expel inhibitory postsynaptic proteins (Zeng et al., 2018). The large complex of multiple scaffold proteins is dependent on multivalent and specific interactions to form LLPS. Therefore, PDZ3 alone cannot form LLPS with SynGap, but requires the PSG supramodule for LLPS formation.

**Figure 2:**
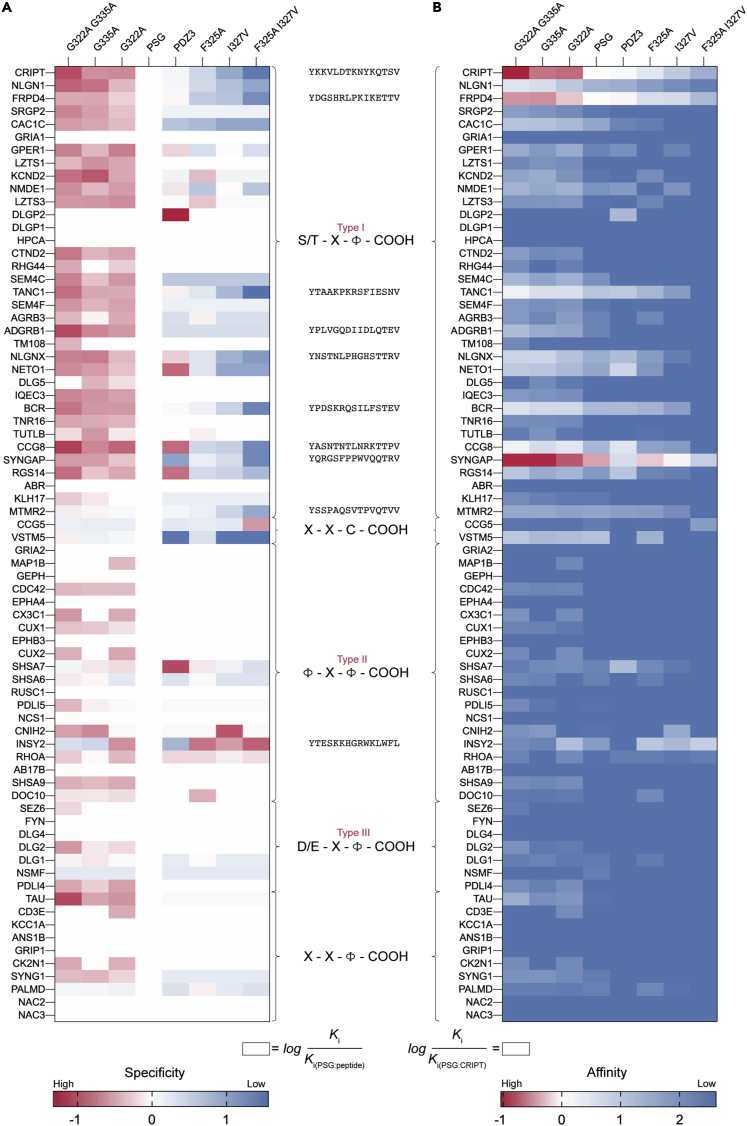
Mapping the specificity profile for mutational PSG variants Heatmap clustered based on known PBM classes as depicted and exemplified on the right *y*-axis. Each pixel represents the binding (*K*_*i*_ from FP) of a 15 AA peptide (row) to a certain PSG variant or to the single domain PDZ3 (column). (A) Each *K*_*i*_ was normalized to the binding of wild-type PSG:peptide, and the log_10_ of this value is reported, such that 0 represents an affinity similar to the PSG:peptide interaction, i.e., log (*K*_*i*_^PSGvariant^/*K*_*i*_^PSG^) = 0. Red represents higher affinity for the PSG variant, log (*K*_*i*_^PSGvariant^/*K*_*i*_^PSG^) < 0, and blue lower affinity for the PSG variant, log (*K*_*i*_^PSGvariant^/*K*_*i*_^PSG^) > 0. As a general trend, pixels in columns with PSG_G322A_, PSG_G335A_, and PSG_G322A G335A_ are red as these proteins have higher affinity than PSG for the peptides. On the other hand, pixels in columns with PSG_F325A_, PSG_I327V_, and PSG_F325A I327V_ are blue or white; thus, these proteins have generally lower or equal affinity for the peptides, as compared with PSG. Based on the heatmap, it is possible to identify shifts in binding specificity. For example, the INSY2-derived peptide reported a shift, as pixels for PSG_F325A_, PSG_I327V_, and PSG_F325A I327V_ are red, whereas pixels for PSG_G335A_ and PSG_G322A G335A_ are blue. A row with only white pixels means that the peptide does not bind to any of the proteins. (B) *K*_*i*_ values were normalized to the *K*_*i*_ of PSG:CRIPT, and the logarithm of this value is shown in the heatmap. Thus, 0 (white) represents an affinity similar to that of the wild-type PSG:CRIPT interaction. Color code: dark blue (low affinity/no binding) and red (higher affinity than wild-type PSG:CRIPT interaction). Row with only blue pixels means that the peptide does not bind to any of the proteins. *K*_*i*_ values were calculated from IC_50_ values determined from fluorescence polarization-monitored displacement experiments performed at room temperature in 50-mM Tris, 100-mM NaCl, 1-mM TCEP, and 0.1% Tween 20 (see Data S1 for numbers).

PDZ is a small (around 100 amino acid residues) protein interaction domain belonging to one of the largest domain families in the human proteome with around 268 members (Christensen et al., 2019). PDZ domains usually bind to the C-terminal (continuous epitope) of other proteins but occasionally also to internal motifs (Mu et al., 2014) and phosphoinositides (Wawrzyniak et al., 2013). PDZ domains are characterized by the C-terminal recognition motif they bind to. Up to 16 distinct specificity classes have been identified in both humans and the nematode *Caenorhabditis elegans*, suggesting that these classes are conserved across bilaterian animals (Tonikian et al., 2008). However, the three most common classes are categorized based on the amino acid at position P_-2_ (Figure 2). The classes have the following PDZ binding motifs (PBMs), where φ denotes a hydrophobic residue and X any residue: type I (T/S-X-φ), type II (φ-X-φ), and type III (D/E-X-φ). PDZ domains are often present in multidomain proteins. Whereas isolated PDZ domains have been thoroughly studied, much less is known about their functional properties and binding specificity in the context of the supertertiary structure, which entails both inter-domain interactions and their dynamics (Tompa, 2012).

In this work, we address general principles that determine the binding affinity and specificity of the PSG supramodule in comparison with PDZ3 alone, by screening 74 putative PDZ binding motifs from proteins present in the PSD (Figure 1A). In general, both PSG and PDZ3 were found to bind with high affinity to peptides with type I PBM, but with different rankings in specificity for the peptides. In particular, PSG has high affinity and specificity to peptides derived from SynGap and ADGRB1, in comparison with PDZ3. Interestingly, ADGRB1, similarly to SynGap, has a high content of coiled coil (CC) and a type I PBM in its C-terminus, and interaction with PSG resulted in LLPS. We also investigated six designed mutational variants of PSG to shed light on the plasticity of the specificity in the PSG supramodule and its capacity to evolve new functions.

## Results

### Library design and construction

We designed a human peptide library containing 74 C-terminal 15 amino acid (AA) peptides (Data S1) from proteins with a potential to bind PDZ domains and which are found in the post-synaptic density (PSD), dendrite, PSD membrane or dendritic spine, as reported in Uniprot (www.uniprot.org). Thus, all proteins with hydrophobic (I, V, L, and F) or cysteine residues at the C-terminal position P_0_ were chosen for the analysis (Figure 1A). Two out of the 74 peptides were previously well characterized for both single domain PDZ3 and the three-domain PSG supramodule: CRIPT, the proposed native ligand of PDZ3, which binds with low μM affinity both to PDZ3 and PSG (Laursen et al., 2020a), and the α_1_ isoform of SynGap (referred to as SynGap), which binds selectively to PSG as interactions outside of the binding pocket in PDZ3 are required for high affinity (Zeng et al., 2016). The PDZ3 interactome has been comprehensively characterized. It shows type I PBM specificity (Niethammer et al., 1998; Saro et al., 2007; Toto et al., 2016), and requires only six amino acids for high-affinity interaction (Saro et al., 2007; Toto et al., 2016), as interactions outside of the binding pocket do not contribute significantly to the affinity. Too few studies have been carried out with PSG to show any trend regarding structural details of its high-affinity interaction (Laursen et al., 2020a; Zeng et al., 2016). To investigate structural determinants of the affinity and specificity, six mutational variants of PSG were included in the study: PSG_G322A_, PSG_G335A_, PSG_G322A G335A_, PSG_F325A_, PSG_I327V_, and PSG_F325A I327V._ The mutations were chosen as they were previously shown to have a significant effect by decreasing (F325A and I327V) or increasing (G322A and G335A) the binding affinity of the native ligand CRIPT (Laursen et al., 2020b).

### Design of fluorescence polarization assay

The characterization of 592 protein:peptide (8 × 74) interactions required a semi-high throughput experimental set-up that can measure weak interactions (high μM) (Lea and Simeonov, 2011; Nikolovska-Coleska et al., 2004). We used a fluorescence polarization (FP) assay for the library screen (Figure 1B), in which a labeled probe peptide was displaced from PDZ3 or PSG by unlabeled peptides. A C-terminal peptide (15 AA, GKKVLDTKNYKQTSV) derived from CRIPT was N-terminally labeled with FITC, thus acting as a probe peptide in the FP assay to characterize new as well as previously known PSG:peptide interactions. The peptide derived from CRIPT was selected as the probe peptide as it has a high affinity for wild-type PSG and was previously shown to bind all PSG variants used in the present study (Laursen et al., 2020b). First, the affinity of the probe (FITC-15 AA CRIPT) was measured for PDZ3 and all seven PSG variants in saturation binding experiments. Twelve different concentrations of each protein variant (PSG, PSG_G322A_, PSG_F325A_, PSG_I327V_, PSG_G335A_, PSG_G322A G335A_, and PSG_F325A I327V_) were incubated with 5 nM of probe (Figures 1B and S1). The binding of FITC-15 AA CRIPT to PSG or PDZ3 was monitored by a change in polarization, as manifested by increased FP signal with increasing protein concentration. The affinity of the FITC-15 AA CRIPT to the PSG variants spanned from 0.16 to 38 μM (Figures 1C and S1) in accordance with previous studies with 15 AA CRIPT (Laursen et al., 2020a, 2020b). The affinities were generally 2- to 4-fold lower than those previously measured by kinetic stopped flow experiments, which can be explained by a different assay set-up with regard to temperature and a different N-terminal modification of CRIPT. The FP assay has several advantages in comparison with stopped flow experiments in terms of high throughput and the possibility to measure low-affinity interactions (*K*_*d*_ ∼10–100 μM). However, the accuracy and precision are limited by the size ratio of the protein:probe, and by the affinity of the probe in displacement screening experiments (see below). For example, a better signal-to-noise ratio was obtained for the larger PSG:probe complex than for the smaller PDZ3:probe. For the low-affinity variant PSG_F325A I327V_, protein solubility hampered complete saturation of FITC-15 AA CRIPT, resulting in a larger variation in the resulting *K*_*d*_ from four independent experiments (Figure S1H).

To determine the affinity of all C-terminal peptides derived from proteins expressed in the PSD, competitive displacement experiments using non-labeled peptide were performed (Figure 1B). Based on the saturation experiments with FITC-15 AA CRIPT (Figure S1), the optimal protein concentration for FP-monitored displacement experiments was chosen for each protein. A good assay window is obtained by a concentration of protein that gives 50–80% saturation of the FP probe (Moerke, 2009). Hence, the concentrations for each protein variant were as follows: PDZ3 (1.3–6 μM), PSG (2–3 μM), PSG_G322A_ (0.5 μM), PSG_G335A_ (0.6 μM), PSG_G322A G335A_ (0.3 μM), PSG_F325A_ (3.8–7 μM), PSG_I327V_ (6–12 μM), and PSG_F325A I327V_ (10–14 μM). We used a concentration of PSG_F325A I327V_ that gave a signal below 50% signal, to reduce the amount of protein required for each experiment.

### Global mapping of ligand affinity and specificity in the PSG protein-protein interaction matrix

Each protein variant was incubated with 5 nM of FITC-15 AA CRIPT, which was then displaced by increasing peptide concentration (2-fold dilution series) of unlabelled 15 AA CRIPT (Figure 1E), 15 AA SynGap (Figure 1F) or one of the other 72 peptides in a 96-well plate format. All protein variants were screened in parallel by using the same peptide concentration series for all eight protein variants, thus allowing for a direct comparison of specificity (relative affinity) for the 74 peptides across the variants. The affinity (*K*_*i*_) was determined from each protein:peptide IC_50_ value using the equation described by Nikolovska-Coleska et al. (Figure 1D) (Nikolovska-Coleska et al., 2004). The FP-determined binding affinities (*K*_*i*_) of all 74 C-terminal peptides derived from proteins in PSD with the respective PSG variant are summarized in Data S1 and visualized in a heatmap (Figure 2). Binding is displayed by a colored pixel representing the affinity between PSG variant:peptide as compared with wild-type PSG:peptide (where white is the same affinity) (Figure 2A). The general trend was that PSG_G322A_, PSG_G335A_, and PSG_G322A G335A_ reported higher affinity in comparison with PSG across all peptides, but especially for peptides with type I PBM, as illustrated by the red color in the heatmap. On the other hand, PSG_F325A_, PSG_I327V_, and PSG_F325A I327V_ displayed lower affinity in comparison with wild-type PSG, as illustrated by the blue color in the heatmap. This map, which depicts the specificity of the PSG variants for each single peptide, does not show whether any of the peptides have a higher affinity for PSG than the native ligand CRIPT. Therefore, in a second heatmap, all PSG variant:peptide interactions were normalized against one interaction, that for PSG: 15 AA CRIPT (Figure 2B). Most interactions were found to have lower affinity than native PSG: 15 AA CRIPT, as shown by the overrepresentation of blue color in the heatmap. Three 15 AA peptides derived from CRIPT, FRPD4, and SynGap bound to PSG_G322A_, PSG_G335A_, PSG_G322A G335A_ with higher affinity than 15 AA CRIPT to wild-type PSG (Figure 2B). One single peptide stands out, SynGap, which bound to PSG and even PSG_F325A_ with high affinity. 15 AA SynGap showed a significantly tighter binding to the PSG supramodule than to PDZ3, likely owing to interactions outside of the binding pocket, as previously reported (Zeng et al., 2016). Furthermore, the mutations in PSG had only little effect on the binding affinity of 15 AA SynGap to PSG. In contrast, PSG_G322A G335A_ displays an approximately 8-fold increase in binding affinity to 15 AA CRIPT compared with PSG. On the other hand, the low-affinity PSG variants, PSG_I327V_ and PSG_F325A I327V_, displayed lower affinity than wild-type PSG for most peptides with few exceptions: INSY2, CNIH2, RHOA, and CCG5 (Figure 2A).

### PDZ3 and PSG have different specificity profiles

Comparison of the binding profiles for PDZ3 and PSG shows similarities and differences. Generally, PDZ3 and PSG bind with high specificity and affinity to all ligands with type I PBM, but we observed differences in the rank of peptides (Figure 2 and Data S1). PDZ3 displayed binding to 10 peptides with *K*_*i*_ below 75 μM listed in order of decreasing affinity: FRPD4, CRIPT, CCG8, SynGap, NETO1, TANC1, RGS14, NLGNX, BCR, NLGN1, and NETO1 (PDZ3). PSG was found to bind eight peptides with *K*_*i*_ below 75 μM: SynGap, FRPD4, CRIPT, TANC1, VSTM5, BCR, CCG8, and NLGN1 (PSG).

### Mutational paths to high and low affinity and new specificity

**Figure 3:**
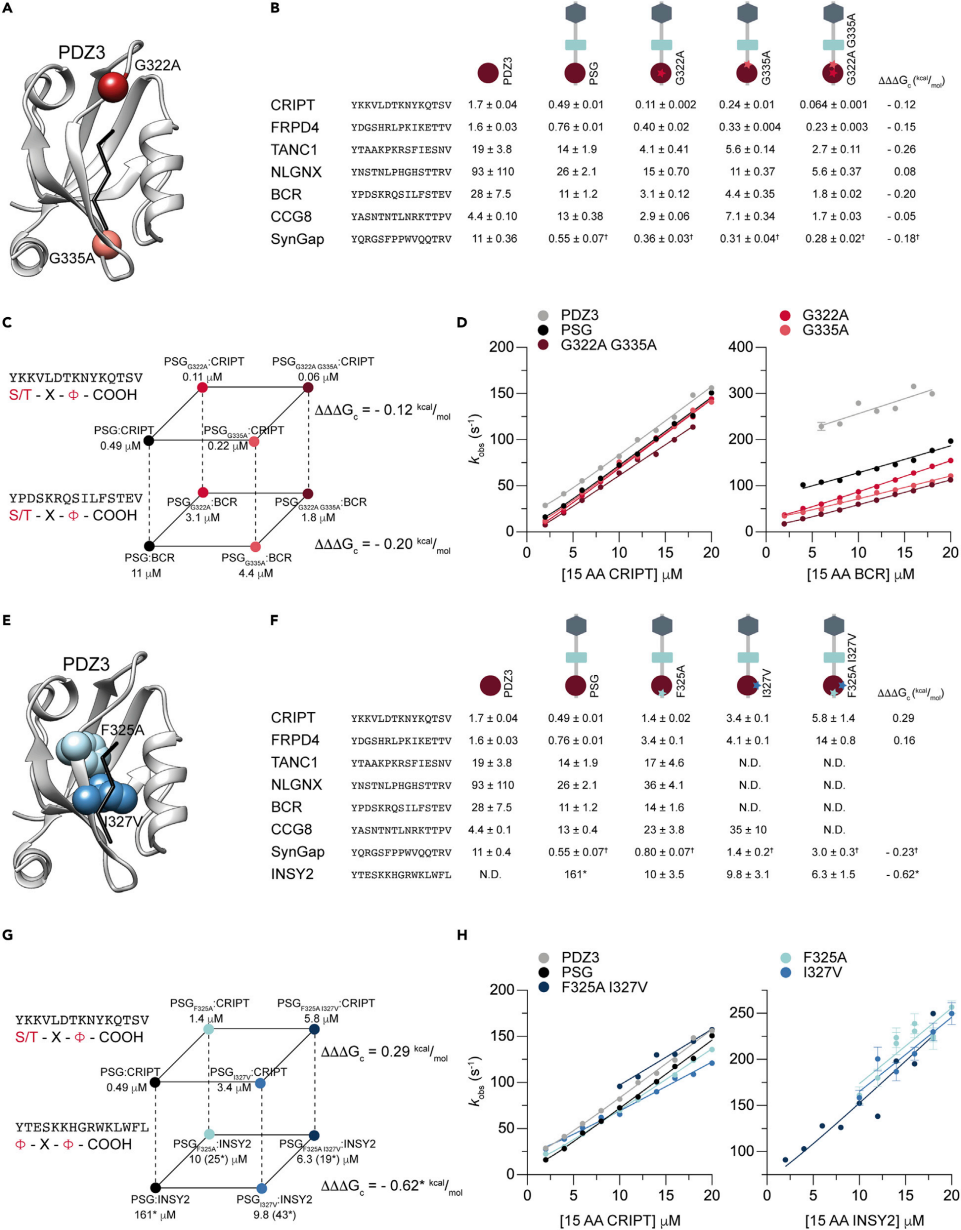
G322A and G335A substitutions promote a selective binding profile for type I PBM while F325A and I327V relax binding motif specificity (A) Residues G322 and G335 are highlighted in the structure of PDZ3 (PDB: 1be9). The backbone corresponding to the five C-terminal amino acids in CRIPT (black) is shown as stick model in the peptide binding pocket (also in E). (B) *K*_d_ values for interactions between PDZ3, PSG, PSG_G322A_, PSG_G335A_, and PSG_G322A G335A_ and seven selected 15 AA peptides from the C-termini of proteins found in the post-synaptic density. Coupling free energies (ΔΔΔ*G*_c_) were calculated from one side square of a thermodynamic cube as depicted in (C). Errors represent propagated fitting errors. (C) A thermodynamic cube showing the effect of G322A and G335A in the context of the native ligand CRIPT (top square) and a potential ligand BCR (bottom square), both with a type I PBM highlighted in red. Both PSG and PSG_G322A G335A_ have an ∼22-– to 28-fold preference for 15 AA CRIPT over 15 AA BCR and, in both cases, the effects of the mutations are additive as shown by the relatively low coupling free energies (B). (D) Observed rate constants from stopped flow experiments were plotted versus increasing concentration of 15 AA CRIPT or 15 AA BCR to obtain the association rate constant (*k*_*on*_) as the slope of the curve. Similar stopped flow experiments were performed for six 15 AA peptides and five PSG variants to get an accurate estimate of binding affinities. (E) Residues F325 and I327 are highlighted in the structure of PDZ3 (PDB: 1be9). (F) *K*_d_ values and coupling free energies for PDZ3, PSG, PSG_F325A_, PSG_I327V_, and PSG_F325A I327V_ and eight selected 15 AA peptides from the PSD. Errors represent propagated fitting errors. (G) A thermodynamic cube showing the effects of F325A and I327V in the context of the native ligand CRIPT (top square) with a type I PBM and INSY2 (bottom square) with type II PBM. Not determined (N.D.) indicates that the affinity was too weak for stopped flow experiments and therefore only determined by fluorescence polarization (Figure 2 and Data S1). ^∗^Affinity determined by ITC owing to complex kinetic binding behavior. ∗Affinity (*K*_*i*_) determined by fluorescence polarization displacement experiment owing to weak interaction precluding stopped flow experiments. *K*_*i*_ from FP and *K*_*d*_ from stopped flow are not directly comparable owing to the experimental setup and could differ by a factor 2–4. (H) Stopped flow-based measurement of interactions with 15 AA CRIPT (left) and INSY2 (right). Observed rate constants are plotted versus increasing concentration of 15 AA CRIPT or INSY2 to obtain the association rate constant (*k*_*on*_) as the slope of the curve. Similar plots were obtained for all eight 15 AA peptides and five PSG variants.

The 268 PDZ domains present in our proteome display a range of overlapping specificities. One key question concerning the evolution of new protein function is how many mutations are necessary to switch specificity in a protein–protein interaction. For example, the affinity for unlabelled 15 AA CRIPT obtained in the displacement experiments spanned from 0.22 to 70 μM among the PSG variants, whereas that of 15 AA SynGap varied between 0.24 and 19 μM, suggesting slight switches in specificity (Figure 2). To further investigate possible specificity switches among the six designed PSG variants (G322A, G335A, G322A G335A, F325, I327, and F325A I327A) we selected eight peptides based on the large-scale screen for more detailed studies: CRIPT, FRPD4, TANC1, NLGNX, BCR, CCG8, INSY2, and SynGap. Seven of the peptides contained a type I motif and one (INSY2) a type II motif. In order to increase the accuracy and precision of the affinity constants for these eight peptides, we determined association (*k*_on_) and dissociation (*k*_off_) rate constants using stopped flow spectroscopy. *K*_d_ is then calculated as the ratio *k*_off_/*k*_on_ (Figure 3 and Data S2). FP was employed for INSY2 when stopped-flow experiments could not be conducted owing to a low-affinity interaction and ITC was used for SynGap (Figure S2) owing to complex binding kinetics. The residues G322 and G335 are situated outside of the peptide-binding pocket (Figure 3A) whereas F325 and I327 make direct contact with the bound peptide (Figure 3E).

The two single mutations G322A and G335A both contributed to increasing the affinity, mainly in an additive fashion, as shown by the low coupling free energy (0 ≥ ΔΔΔ*G*_c_ ≥ −0.3) calculated from affinity data for the four variants PSG, PSG_G322A_, PSG_G335A_, and PSG_G322A G335A_ (Figures 3A–3D). On the other hand, the two mutations F325A and I327V generally decreased the affinity for the peptides, except for the type II PBM peptide from INSY2, which displayed enhanced affinity (Figures 3E–3H). We could estimate the affinity for all four PSG variants (wild-type PSG, PSG_F325A_, PSG_I327V_, and PSG_F325A I327V_) for four of the peptides (CRIPT, FRPD4, SynGap, and INSY2) using a combination of stopped flow, ITC, and FP. This enabled calculation of ΔΔΔ*G*_c_ values between the side chains of F325 and I327 during interaction with the respective peptide (Data S2). Three of the four peptides (CRIPT, FRPD4, SynGap) reported ΔΔΔ*G*_c_ values around −0.2 to 0.3 kcal/mol, meaning that F325 and I327 do not have a strong synergistic effect on binding. However, INSY2 stood out with ΔΔΔ*G*_c_ = −0.62 kcal/mol suggesting a negative cooperative effect between F325 and I327 upon interaction with the type II motif peptide from INSY2. In other words, the first mutation attenuates the effect of the second mutation. Of all type II peptides in the screen, only INSY2 was identified as a binder, even though two peptides from other proteins, CUX1 and CUX2, had a large hydrophobic group at the P_-2_ position. CUX1 and CUX2 have a W-X-F binding motif, but large hydrophobic residues both at positions P_0_ and P_-2_ are rare for canonical binding of C-terminal peptides to PDZ domains (Tonikian et al., 2008). PDZ domains that bind to internal peptides can bind to peptides with Phe at the P_0_ position, possibly because they have a more plastic binding pocket (Christensen et al., 2019; Penkert et al., 2004).

### Correlation between phase separation and affinity

**Figure 4:**
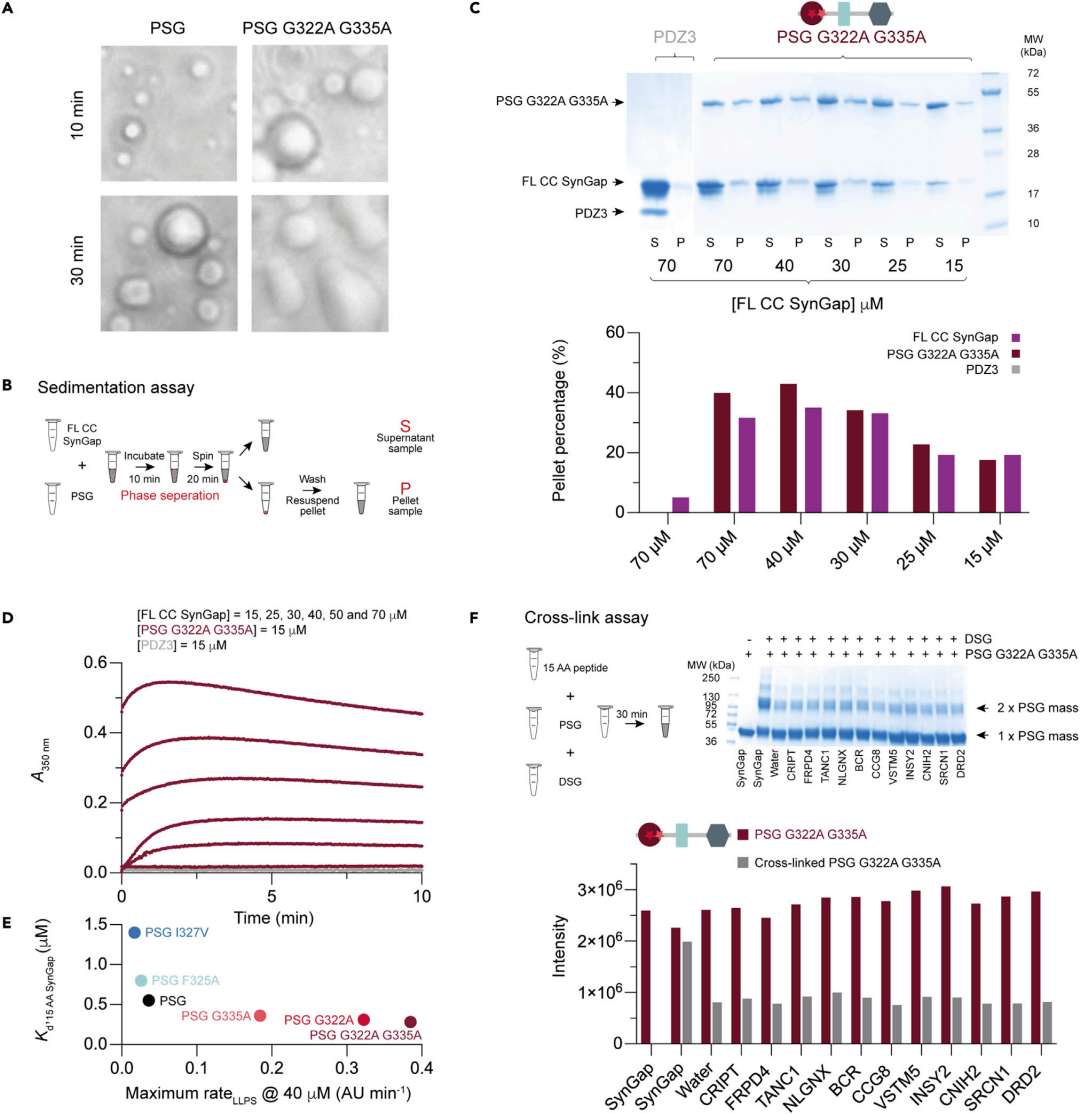
Affinity between FL CC SynGap and PSG variants is correlated with phase transition properties (A) Light microscopy at room temperature: mixing of PSG or PSG_G322A G335A_ and FL CC SynGap in a 1:3 ratio resulted in the formation of small liquid droplets that merged over time and formed larger droplets. (B) Illustration of the sedimentation assay used to quantify the amount of LLPS formation, as represented by the pellet sample. (C) Bar graph diagram (bottom) showing the quantification of the sedimentation assay analyzed by band intensity from SDS-PAGE (top). Pellet percentage quantifies the amount of LLPS formation at each concentration of FL CC SynGap. No LLPS was observed for PDZ3, whereas the LLPS formation of FL CC SynGap:PSG_G322A G335A_ was concentration-dependent. (D) The kinetics of liquid-liquid phase separation was monitored by change in turbidity of the solution, as measured by absorbance at 350 nm. PSG_G322A G335A_ or PDZ3 concentration was constant (15 μM) and challenged with different concentrations of FL CC SynGap. The rate of LLPS formation, as measured by turbidity, correlates with increasing FL CC SynGap concentration for PSG_G322A G335A_, whereas no LLPS formation was observed for PDZ3 or PSG_F325A I327V_ in the chosen concentration range of FL CC SynGap. (E) The observed maximum rate of LLPS formation at 40-μM FL CC SynGap was obtained from analysis of the kinetic traces in (D) and Figure S3. The maximum rate for LLPS formation and the affinity for 15 AA SynGap are clearly correlated for the PSG variants. (F) Disuccinimidyl glutarate (DSG) mediated cross-link assay to test if the 15 AA peptides can induce dimerization. SDS-PAGE of DSG mediated cross-linking reveals that 15 AA SynGap is the only of the 12 peptides that can induce dimerization of PSG_G322A G335A_. The bar diagram shows SDS-PAGE quantification of dimer (gray) in comparison with monomeric PSG_G322A G335A_ (bordeaux).

It was previously shown that PSG and a CC fragment of SynGap, denoted “full-length” (FL) CC SynGap form LLPS (Zeng et al., 2016). Generally, condensates resulting from LLPS are important for membraneless spatial organization (Li et al., 2012), biomolecular reaction centers, signaling hubs, and support architecture inside the cell (Zhang et al., 2020). PSG variants display a range of affinities toward peptide ligands including a 10-fold difference for 15 AA SynGap (Data S2, Figures 3 and S2). To explore how affinity modulates LLPS, the PSG variants were examined using different assays including sedimentation and turbidity experiments (Figures 4 and S3). First, we mixed PSG WT or PSG_G322A G335A_ with FL CC SynGap and observed the formation of LLPS under the light microscope (Figure 4A). Liquid droplets evolved from a spherical shape to a more elongated shape over time. The elongated shape was most profound for the PSG_G322A G335A_:SynGap complex, probably owing to a lower LLPS threshold for PSG_G322A G335A_ as compared with PSG WT, which in turn depends on the affinity for SynGap. Therefore, it seems like droplets start to merge into elongated shapes owing to the crowded environment (30 min, PSG_G322A G335A_:SynGap). Next, we performed a sedimentation assay (Figure 4B) in which the fraction of LLPS was quantified by SDS-PAGE (Figures 4C and S3). Indeed, higher concentrations of PSG variant and FL CC SynGap resulted in more LLPS. We observed a discrepancy for PSG_F325A_ in the sedimentation assay, in which a larger fraction of the protein is observed in the pellet fraction than expected from the turbidity assay and affinity (Figure S3C). A likely explanation is that this variant is unstable over time and precipitates at high protein concentrations.

Finally, in an attempt to assess the kinetics of LLPS we mixed PSG variants with different concentrations of FL CC SynGap and followed the increase in absorbance at 350 nm over time (Figures 4D and S3). At this wavelength, an increase in absorbance results from light scattering, or turbidity, which can be anticipated to correlate with LLPS. The kinetics were multiphasic including a lag phase showing that the LLPS formation process is complex, as can be expected. Nevertheless, the kinetic transients show that the maximum rate of LLPS formation, occurring between the lag phase and the stationary phase, increases with FL CC SynGap concentration (Figures 4D and S3). At higher FL CC SynGap concentrations, and depending on the PSG variant, the initial binding event(s) occurred too fast to be captured in our experiments as shown by the high absorbance at time zero in the time courses. The observed maximum rate appeared to increase linearly with FL CC SynGap concentration, consistent with a molecular association event (Figure S3F). A comparison of the maximum rate of LLPS between the PSG variants at 40 μM FL CC SynGap shows a clear correlation where the higher-affinity variants PSG_G322A_, PSG_G335A_, and PSG_FG332A G335A_ displayed a higher maximum rate of LLPS (Figure 4E).

In conclusion, the data on LLPS (Figures 4 and S3) and affinity of SynGap for PSG variants (Figure S2) show that LLPS formation correlates with affinity (Figure 4E). This conclusion is supported by light microscopy-monitored liquid droplet formation at different time points for PSG:FL CC SynGap and PSG_G332A G335A_:FL CC SynGap (Figure 4A).

### ADGRB1 shows LLPS with PSG

It was previously reported that the C-terminal peptide of SynGap induced dimerization of PSG (Zeng et al., 2016). Such dimerization could act as a proxy for phase separation. Therefore, in an attempt to identify new proteins that phase separate with PSD-95, 10 high-affinity ligands identified on the screen were examined for their potential to induce dimerization of PSG (Figure 4F). Thus, PSG was mixed with each of the ten different 15 AA peptides and with the cross-linker disuccinimidyl glutarate (DSG) for 30 min (Zeng et al., 2016). The amount of crosslinked PSG was quantified by SDS-PAGE. However, all peptides except SynGap (positive control) showed the same amount of PSG cross-linking as afforded by CRIPT (negative control) or water (Figure 4F), suggesting that dimerization is a unique property induced by SynGap (Zeng et al., 2018). Whereas dimerization is not required for LLPS (Mitrea and Kriwacki, 2016), it will increase multivalent interactions, which seems to be important for a low LLPS threshold (Christensen et al., 2022; Zeng et al., 2018).

To further investigate LLPS involving putative native ligands for PSD-95 we employed bioinformatics to find proteins with potential for phase separation. LLPS is associated with a high propensity of forming a CC (Ford and Fioriti, 2020; Zhang et al., 2020) as observed for a sequence stretch of 60 amino acids in SynGap. We calculated the CC propensity for all PSD proteins included in the study using the COILS software (Lupas et al., 1991) (Figure S4). CRIPT was found to have a low CC propensity, which is in line with the finding that CRIPT does not form LLPS with PSG (Zeng et al., 2016). Out of the 74 PSD proteins on the screen, 9 proteins were found to have a high propensity for CC formation (Figure S4), but only three of them showed an affinity corresponding to a *K*_d_ < 100 μM, even to the high-affinity PSG_G322A G335A_ variant (Figure 2, Data S1). Myotubularin-related protein 2 (MTMR2) that interacts with PSD-95 to maintain excitatory synapses by modulating endosomal traffic (Lee et al., 2010) and Adhesion G protein-coupled receptor B1 (ADGRB1) were chosen for further analysis, as they combine a high propensity of forming a CC with *K*_*i*_ values from the FP-based assay of 95 and 39 μM, respectively, to PSG_G322A G335A_. Indeed, ADGRB1, but not MTMR2, showed LLPS in initial test studies.

**Figure 5:**
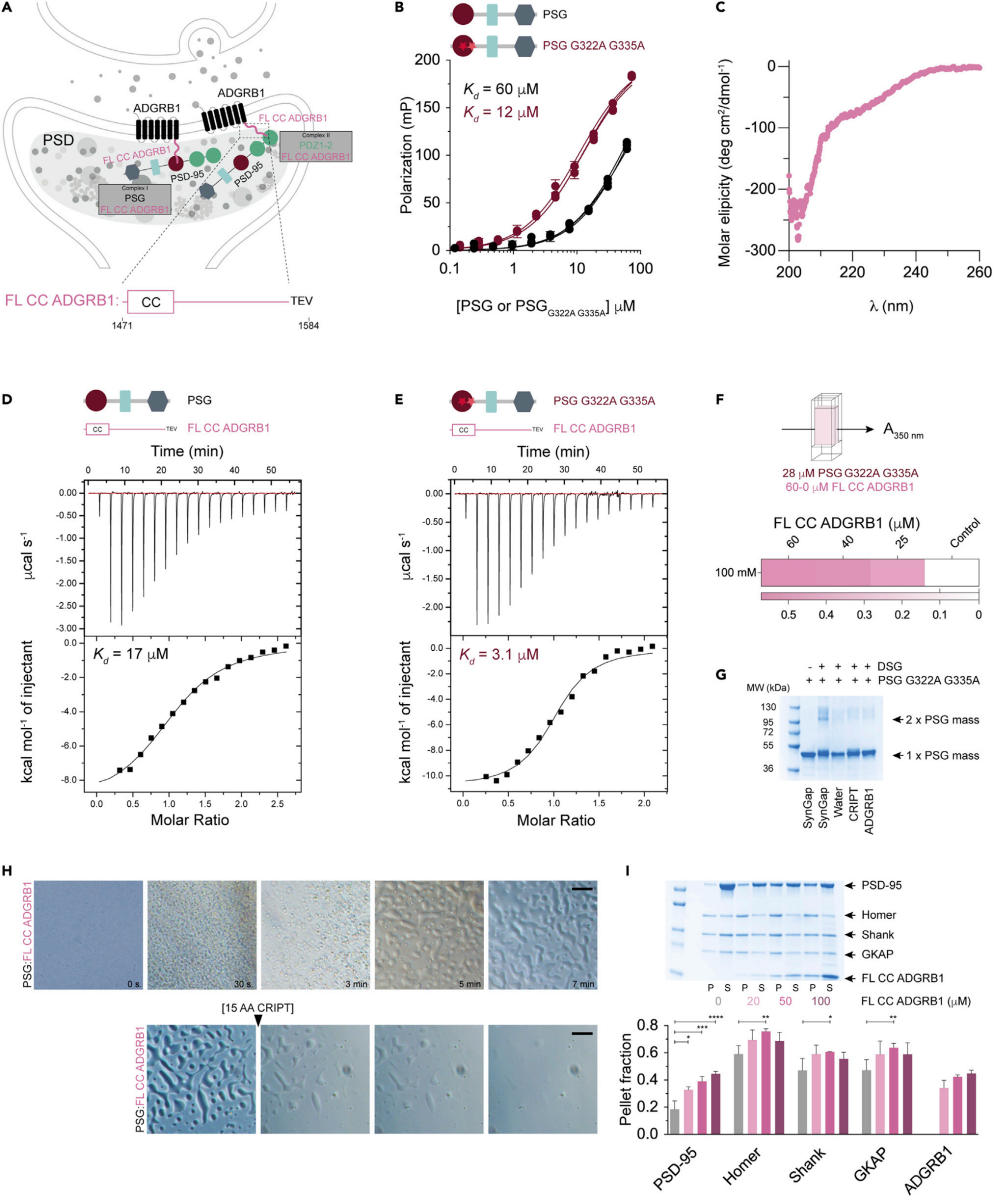
Phase transition of PSD-95 PSG:FL CC ADGRB1 complex (A) Schematic representation of a synapse highlighting the postsynaptic neuron were ADGRB1 receptors are located in the postsynaptic membrane. The C-terminal part of ADGRB1 (pink) is intracellular and located next to the PSD. The PSG supramodule of PSD-95 is highlighted to illustrate the possible interaction between PSG and full-length coiled-coil C-terminal part of ADGRB1. (B) Fluorescence polarization-based measurement of the PSG:ADGRB1 interaction. Data were obtained by titration of FITC-14 AA ADGRB1 with PSG or PSG_G322A G335A_, respectively. The experiments were performed at room temperature in technical triplicates and biological duplicates. (C) Analysis of FL CC ADGRB1 by circular dichroism between 200 and 260 nm suggests it is a disordered protein. The spectrum is an average of five scans measured at 25°C in 100-mM NaCl, 50-mM Tris pH 7.8, 1-mM TCEP. (D and E) ITC experiments of the interaction between PSG and FL CC ADGRB1 (D) and PSG_G322A G335A_ and FL CC ADGRB1 (E). (F) LLPS formation measured as turbidity over time by monitoring absorbance at 350 nm at 25°C. PSG_G322A G335A_ at a constant concentration of 15 μM was mixed with different concentrations of FL CC ADGRB1. LLPS formation correlates with the increasing FL CC ADGRB1 concentration. (G) SDS-PAGE of DSG-mediated cross-linking shows that 14 AA ADGRB1 cannot induce dimerization of PSG_G322A G335A_. 15 AA SynGap is a positive control and 15 AA CRIPT and water are negative controls. (H) LLPS of PSG and FL CC ADGRB1. Isolated PSG and FL CC ADGRB1 are soluble and homogeneous at 200 μM, as judged by light microscopy at room temperature. Mixing of PSG and FL CC ADGRB1 in a 1:3 ratio resulted in the formation of small liquid droplets that merged over time and formed larger droplets. The large liquid droplets formed by PSG and FL CC ADGRB1 immediately started to disappear upon the addition of 15 AA CRIPT. (I) SDS-PAGE sedimentation assay where PSD-95, Homer, Shank, and GKAP (each 20 μM) were incubated with increasing concentration of FL CC ADGRB1. Higher concentration of ADGRB1 clearly correlates with higher inclusion of PSD-95 in the pellets representing LLPS, whereas a correlation of lower significance was observed with Homer, Shank, and GKAP. Data were quantified by Lab software from BioRad. The significance (∗*p* < 0.05; ∗∗*p* < 0.01; ∗∗∗*p* < 0.001) was evaluated by two-way ANOVA with Tukey test using Prism 9.0 software (GraphPad).

ADGRB1 is a seven transmembrane helix protein highly enriched in the postsynaptic membrane with an N-terminal extracellular and a C-terminal intracellular portion (Figure 5A). The protein is suggested to regulate Rho signaling through a PDZ:ADGRB1 interaction (Stephenson et al., 2013). The C-terminal part (residues 1,471–1,584) of ADGRB1 is anchored into the PSD, thus, PSD-95 PSG and the C-terminus of ADGRB1 is spatially located such that a physical interaction in the postsynaptic neuron is possible. We therefore subjected this interaction to further experiments. A direct FP-monitored binding experiment with a FITC-14 AA ADGRB1 peptide confirmed the binding to PSG and PSG_G322A G335A_ (Figure 5B). Furthermore, we observed weaker binding of the ADGRB1 peptide to the single domain PDZ3 constructs, PDZ3, PDZ3_G322A_, and PDZ3_G335A_ (Figure S5), showing that binding is more specific for the PSG supramodule.

Next, FL CC ADGRB1 (residues 1,471–1,584) was expressed and purified. The far-UV circular dichroism spectrum of FL CC ADGRB1 displayed a minimum shift toward 200 nm rather than the 208 nm expected for an alpha helix, and the second expected minimum at 222 nm was not present (Figure 5C). Thus, FL CC ADGRB1 has low secondary structure content, as also predicted by the Alpha-Fold 3D model of ADGRB1 (Jumper et al., 2021; Varadi et al., 2022) and suggested from a previous study with the C-terminal part of ADGRB1 (Weng et al., 2019). The binding of FL CC ADGRB1 to PSG and PSG_G322A G335A_ was confirmed with isothermal titration calorimetry (Figures 5D and 5E).

We then performed the DSG-based crosslinking assay to characterize if ADGRB1 induces dimerization of PSG, like SynGap (Zeng et al., 2016). However, this appears to not be the case, as the gel bands with crosslinked PSG are weak for ADGRB1 in comparison with SynGap, and more similar to the controls, water, and CRIPT (Figure 5G). PSD-95 has been reported to undergo LLPS with both SynGap and Stg, however in different molar ratio, 2:3 (Zeng et al., 2016) and 1:1 (Christensen et al., 2022), respectively. Thus, based on the DSG assay and ITC we expected a 1:1 ratio for the ADGRB1:PSG complex. To further address the stoichiometry, we attempted SEC-MALS experiments with PSG and FL CC ADGRB1. However, it was not possible to perform experiments with the protein complex likely owing to phase separation or aggregation at the desired concentration range.

To explore whether FL CC ADGRB1 could phase separate with PSG, like SynGap, we performed light microscopy experiments. Interestingly, the mixing of PSG and FL CC ADGRB1 resulted in the appearance of many small liquid droplets (Figure 5H). Over time, the small droplets assembled into larger ones. After 7 min, an excess of 15 AA CRIPT was added, resulting in the disappearance of the liquid droplets within seconds. The most likely explanation is that the PSG:FL CC ADGRB1 complex was disrupted by competition, where 15 AA CRIPT replaced FL CC ADGRB1 in the complex. This experiment showed that PSG and FL CC ADGRB1 form reversible LLPS, similarly to the interaction between PSG and FL CC SynGap (Zeng et al., 2016).

In an attempt to quantify the LLPS formation, PSG_G322A G335A_ was mixed with FL CC ADGRB1 and turbidity was monitored over time, at increasing concentrations of ADGRB1 (Figure 5F) or NaCl (Figure S6B). The turbidity assay was used to monitor the phase separation of the PSG_G322A G335A_:FL CC ADGRB1 complex by titrating increasing concentration of FL CC ADGRB1 (0–60 μM) into a PSG_G322A G335A_ solution (28 μM, 100-mM NaCl) and monitoring the light scattering at 350 nm over 10 min. The maximum absorbance is reported against increasing FL CC ADGRB1 or NaCl concentrations. Indeed, PSG_G322A G335A_:FL CC ADGRB1 LLPS is concentration- (Figure 5F) and salt-dependent (Figure S6B), similarly as found for SynGap (Figures 4C, 4E, and S6A) and as previously reported for other protein complexes that induce LLPS (Bai et al., 2021; Zeng et al., 2016). The hypersensitivity to the salt concentration of protein complexes undergoing phase separation has been suggested as a mechanism to regulate protein clustering in neurons by activity-induced ion influx from the synapse (Bai et al., 2021).

Finally, we determined the interaction between ADGRB1 and other PSD proteins with regard to phase separation by using the sedimentation assay. The PSD contains several scaffold proteins and the four most abundant ones are PSD-95, GKAP, Homer, and Shank. These proteins can all undergo LLPS and incorporate other PSD proteins such as SynGap and NR2B (Zeng et al., 2018). Indeed, we found that ADGRB1 could undergo LLPS and be incorporated in a reconstituted PSD, similarly to SynGap (Figure 5I).

### Multivalency decreases the threshold for phase separation

All three PDZ domains from PSD-95 (PDZ1, PDZ2, and PDZ3) have an overlapping interactome and bind to proteins with type I PBM (Lim et al., 2002). We therefore investigated whether PDZ1 and PDZ2 could also interact with ADGRB1, and compared the affinity with that of SynGap (Figure S7). Binding of FL CC ADGRB1 (Figure S7B) and 14 AA ADGRB1 (Figure S7C) to a PDZ1-2 tandem construct was determined and showed a 3-fold higher affinity in comparison with PSG WT (Figures 5B and 5D), suggesting that the ADGRB1 interaction is not specific for PDZ3 in the PSG supramodule. To further characterize the PDZ1-2:ADGRB1 complex we investigated whether PDZ1-2 could induce phase separation with FL CC ADGRB1. Interestingly, phase separation with FL CC ADGRB1 was not observed for PDZ1-2 under the same conditions as used for PSG (Figure S7D). Most known PSD-95 complexes have a 1:1 stoichiometry, whereas that between SynGap and PSD-95 has a 3:2 stoichiometry (Zeng et al., 2016). Thus, we performed SEC and SEC-MALS with FL CC ADGRB1 and PDZ1-2 and found that data were most consistent with a 1:1 complex (Figures S7E and S7F). To corroborate our result, we reconstituted the PSD both with PSD-95 or PSG and PDZ1-2 (Figures 5I and S8). PSG can form LLPS in the absence of ADGRB1 and SynGap in a multivalent complex with GKAP, Shank, and Homer (4xPSD) even though its *K*_*d*_ for GKAP (Zeng et al., 2018) is 10 times higher than the final concentration of proteins in the reconstituted PSD (Figure S8C). PDZ1-2 is only enriched in the PSD condensates (Figure S8B and S8C) at a final concentration of FL CC ADGRB1 ten times above *K*_*d*_ (Figure S7B). In conclusion, SynGap is specific for PSG and induces LLPS, whereas ADGRB1 binds to both PSG and PDZ1-2 with similar affinity, but the threshold for LLPS is significantly lower for PSG than for PDZ1-2 (Figure S7G).

### PSG and ADGRB1 form LLPS in living cells

**Figure 6:**
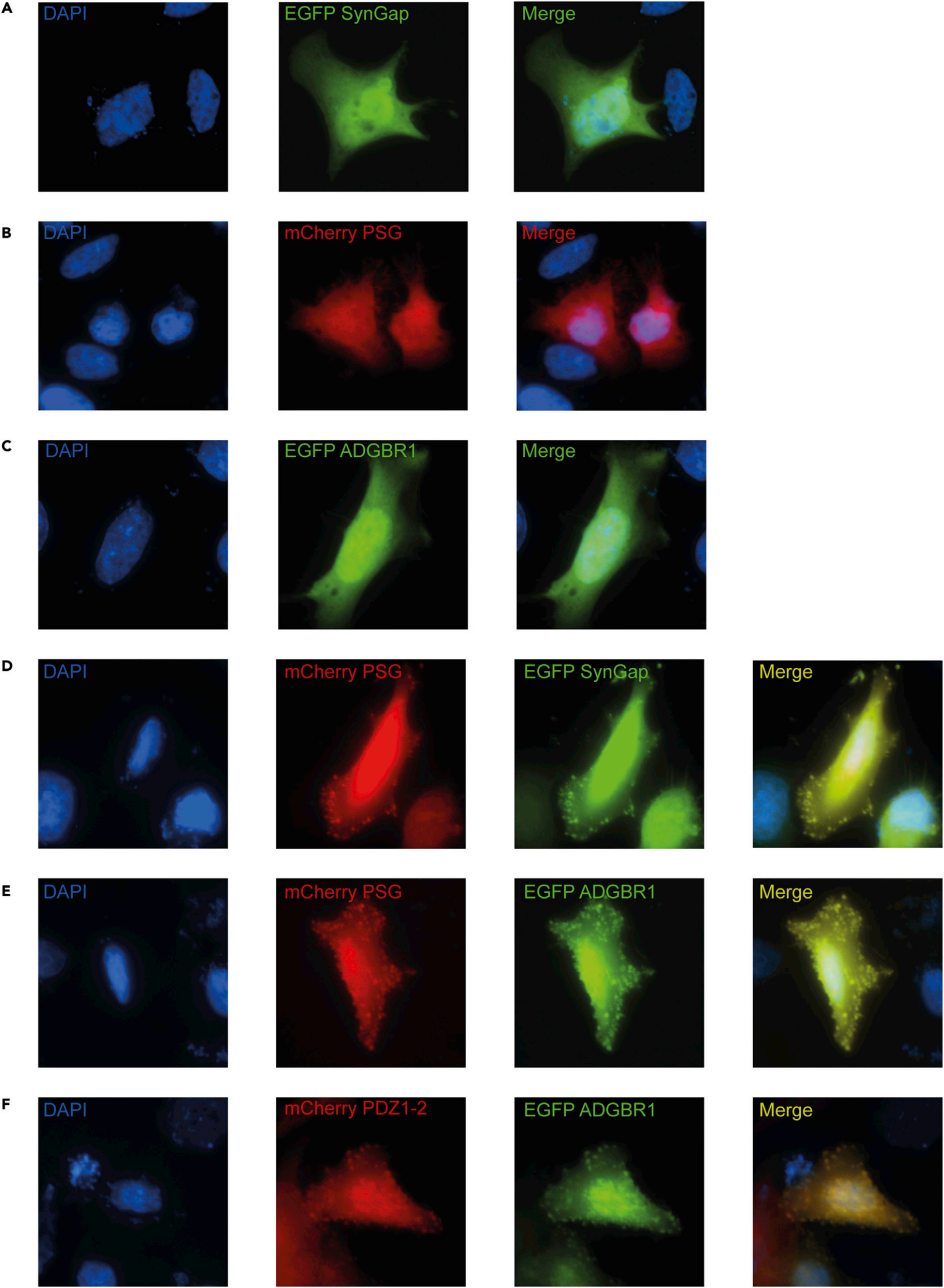
PSG and ADGRB1 form liquid-liquid phase separation in living cells Representative fluorescence microscopy images showing co-localization of PSG and ADGRB1 proteins in HeLa cells. DAPI-stained nucleus, mCherry-stained red cells, and EGFP-stained green cells along with a merge of the images are shown. (A–C) Expression of EGFP-FL CC Syngap, mCherry-PSG and EGFP-FL CC ADGRB1, respectively. (D and E) Co-localization of (D) EGFP-FL CC Syngap and (E) EGFP-FL CC ADGRB1 with mCherry-PSG. (F) Co-localization of EGFP-FL CC ADGRB1 with mCherry-PDZ1-2.

Next, we tested if PSG and FL CC ADGRB1 display co-localization and undergo a phase transition in HeLa cells (Figure 6). As negative control experiments, EGFP-FL CC SynGap (Figure 6A), mCherry-PSG (Figure 6B), and EGFP-FL CC ADGRB1 (Figure 6C) were individually expressed in HeLa cells, resulting in a uniform distribution of EGFP and mCherry signal. However, when EGFP-FL CC SynGap and mCherry-PSG were co-expressed, many bright puncta with both EGFP and mCherry were observed (Figure 6D), recapitulating previous experiments and suggesting that SynGap and PSG were enriched in these puncta. Thus, this experiment serves as a positive control and is consistent with the formation of a condensed liquid phase in living cells, as previously demonstrated (Zeng et al., 2016). When EGFP-FL CC ADGRB1 and mCherry-PSG (Figure 6E) or mCherry-PDZ1-2 (Figure 6F) were co-expressed in HeLa cells many bright puncta with co-localization of both GFP and mCherry signal were observed. These data suggest that ADGRB1, similarly to SynGap (Figure 6D), can co-localize and bind to PSD-95. Like for SynGap, this co-localization and the LLPS directly observed in the light microscope (Figure 5H) indicate that ADGRB1 could form a condensate with PSD-95 in the cell.

## Discussion

### Single domains versus supertertiary structure – similar but different

Interaction between two proteins is often mediated via a folded protein domain in one protein and a short linear binding motif in a disordered region of the other one. PDZ domains and their binding to C-terminal sequences provide a typical example of such types of interaction. The majority of all large-scale screens used to identify potential protein-protein interactions use isolated protein domains. However, it is clear that supertertiary structure, *i*.e., structure and dynamics involving several interacting domains (Tompa, 2012), affects the function of multidomain proteins. We have previously shown that supertertiary structure of the PSG supramodule affects allostery in the PDZ3 domain (Laursen et al., 2020b) and folding of the supramodule (Laursen et al., 2021). In the present study, we found that supertertiary structure also influences the interactome of PDZ3, such that distinct specificity profiles are observed for PDZ3 and PSG. More specifically, whereas both PDZ3 and PSG prefer binding of peptides with a type I PBM, they differ in specificity, *i.e*., the ranking of peptide ligands based on affinity. In particular, SynGap and ADGRB1 bind with significantly higher affinity to the PSG supramodule than to single domain PDZ3. Furthermore, LLPS involving SynGap and ADGRB1 requires the supramodule PSG. Previous experimental and computational studies, reviewed by Feng and Zhang (2009), are consistent with our experimental results, highlighting that multidomain proteins should be examined both at the individual and multidomain level, owing to target-binding properties that differ from those of individual domains. Multiple studies show that stability (Kirubakaran et al., 2016; Vishwanath et al., 2018), folding pathway (Laursen et al., 2021; Vishwanath et al., 2018), allostery (Kirubakaran et al., 2016; Laursen et al., 2020b), binding affinity (Vistrup-Parry et al., 2021; Zeng et al., 2016), LLPS (Vistrup-Parry et al., 2021; Zeng et al., 2016), and the effect of phosphorylation (Vistrup-Parry et al., 2021) can be affected, when comparing single domain with multiple domain proteins. Here we observe that mutations in PDZ3, as part of PSG, can significantly alter the affinity of CRIPT, whereas the same mutations have a minor effect on the binding of SynGap, likely because non-canonical interactions contribute to the affinity. We also show that FL CC ADGRB1 and FL CC SynGap can undergo LLPS with PSG, but LLPS cannot be induced with PDZ3 alone as multiple interactions are essential. These examples underscore the importance of studying protein interactions in the context of their supertertiary structure. The study of multidomain proteins that induce LLPS will further allow us to reveal the function of allosteric signaling. Decades of allosteric network analysis in PDZ3 indicate that the network is dependent on the context (Gautier et al., 2018), as different networks have been reported in PDZ3 (Gianni et al., 2011), PDZ3Δα_3_ (Faure et al., 2022; McLaughlin et al., 2012), and PSG (Laursen et al., 2020b). This sensitivity suggests that the allosteric signaling in protein domains will be affected by the crowded milieu in the cell and by LLPS (Kuznetsova et al., 2015), which is further discussed in a recent review by Nussinov et al. (2022).

### Change of specificity in PDZ domains

Emergence of new protein–protein interactions frequently occurs during evolution, and the large number of PDZ domains in our proteome demonstrates the versatility of this domain as an interaction module. It is of interest to understand how many mutations are necessary to change specificity in protein interactions. We applied double mutant cycle analysis to measure coupling free energy (ΔΔΔ*G*_c_), and thereby identify residues that are allosterically coupled (Horovitz and Fersht, 1990). We found a high coupling free energy between F325 and I327 upon binding to INSY2, paralleled by a switch in specificity, from a 67-fold preference for CRIPT over INSY2 (for wild-type PSG), to no discrimination between the peptides (for the double mutant PSG_F325A I327V_). The structural basis for this shift from type I toward type II specificity is likely that F325A and I327V make space for the binding of peptides with a large hydrophobic residue at the P_-2_ position in the binding motif. In a previous study, two other mutations in the binding pocket of PDZ3, G330T and H372A, switched specificity from type I to a type II motif (F-X-φ) (Raman et al., 2016). The authors showed that the G330T mutation increased the conformational plasticity of the binding pocket resulting in the conditionally neutral variant PDZ3_G330T_ with similar affinity for type I and type II PBMs, whereas the H372A mutation switched the specificity. Similarly, the present mutations of residues F325 and I327 in the peptide-binding pocket of PDZ3 in PSG can be described as conditionally neutral or class bridging, as the mutations preserve the original function (binding CRIPT), but also open up for a new function by binding to a type II motif peptide. Obviously, different mutational routes with few mutations can switch specificity from type I to type II in PDZ domains.

### Multivalent ligands induce phase separation of scaffold proteins

PDZ3 did not show high specificity and affinity for any particular C-terminal peptide, suggesting that the specificity of PDZ3 is plastic as long as the peptide has a type I PBM. On the other hand, a high-affinity interaction was reported between PSG and SynGap, suggesting that context, *i.e*., interactions outside of the canonical binding site, shapes domain-peptide interactions (Li et al., 2012), which is important for PDZ domains in scaffold proteins to gain high affinity (Erlendsson et al., 2019) and specificity in a cellular context (Zeng et al., 2016). Comparison of CRIPT and SynGap reveals that both ligands have type I PBM, but only SynGap can be described as a multivalent ligand as interactions outside of the binding pocket contribute significantly to the high affinity of SynGap (Zeng et al., 2016). Multivalency is an important factor for stabilization of LLPS, an ability that monovalent ligands like CRIPT lack. Indeed, monovalent interactions will destabilize LLPS (Ruff et al., 2021) as we demonstrated by the addition of CRIPT to liquid droplets formed by PSG and ADGRB1. A computational study reporting the effect of ligand type on phase separation revealed that LLPS can be induced under the saturation limit of the scaffold protein if a multivalent ligand is present (Ruff et al., 2021). In our study, we observed such behavior for the multivalent ligand FL CC ADGRB1. These results suggest that the multiple interactions formed between PSG and FL CC ADGRB1 are important to boost affinity and achieve a low LLPS threshold. The conclusion is further supported by all the experimental studies with SynGap and PSD-95 (Zeng et al., 2016, 2018) and a recent study, where LLPS with tri-Stg was observed for FL PSD-95 at 36 μM but only at a higher concentration (256 μM) for PDZ1-2 (Christensen et al., 2022). All together the experimental and computational studies show that scaffold proteins can induce LLPS at a concentration threshold lower than its saturation limit in the presence of a multivalent ligand, an important finding for LLPS *in vivo*, when the endogenous concentration of scaffold proteins can be below the saturation limit (Ruff et al., 2021).

### Phase transition of the PSG:ADGRB1 complex and its implication for anchoring of PSD to the membrane bilayer

We identified ADGRB1 as a potential ligand for PSD-95. Decreased levels of ADGRB1 are reported in patients diagnosed with Parkinson’s disease (Choi et al., 2018; Zhong et al., 2020). Overexpression of ADGRB1 resulted in decreased levels of nuclear condensations associated with cell death and Parkinson's disease, suggesting that ADGRB1 can prohibit cell death in the neurodegenerative pathway (a potential therapeutic target) of Parkinson’s disease (Choi et al., 2018). In a previous study, ADGRB1 was suggested to prevent polyubiquitination and degradation of PSD-95 through an interaction with the ubiquitin ligase MDM2 (Zhu et al., 2015). An ADGRB1 knockout mouse showed a thin PSD and decreased level of PSD-95, which could be rescued by PSD-95 gene therapy, suggesting synaptic plasticity and a reversible modulation of PSD-95 and PSD through ADGRB1. The indirect interaction between PSD-95 and ADGRB1 was demonstrated by a C-terminal truncation of the PBM (QTEV) in ADGRB1. The truncation decreased the PSD-95:ADGRB1 interaction, but preserved the level of PSD-95 suggesting that modulation of PSD-95 by ADGRB1 is through MDM2 and not through a direct interaction. In the present study, we demonstrate a direct interaction between ADGRB1 and PSD-95, which could even promote LLPS, suggesting a new potential mechanism for PSD to anchor to the transmembrane bilayer. It is clear that multiple interactions are required for full affinity between PSD-95 and ADGRB1, as the coiled-coil domain binds in synergy with the canonical PDZ-binding motif. LLPS is dynamic and reversible, but specific mutations and post-translational modifications will hamper the reversibility and induce protein aggregation, which is a common tell-tale of neurodegenerative diseases. New drug development for neurodegenerative diseases aims to maintain LLPS by suppressing mutations and post-translational modifications that cause aggregation of proteins (Wang et al., 2021). Further studies are required to establish the interplay between PSD-95, ADGRB1, and MDM2 and their function in LLPS and protein aggregation.

LLPS currently emerges as a very important property of proteins underlying several functions in the living cell (Alberti and Hyman, 2021). Phase separation facilitates spontaneous and rapid compartmentalization, such as the LLPS induced by the PSD-95:SynGap complex, which is associated with the formation of the PSD (Zeng et al., 2016). LLPS in PSD formation is supported by experiments where the PSD was reconstructed and the concentration threshold for phase separation of PSD-95 and SynGap was reduced by the addition of three other PSD-95 associated scaffolding proteins: GKAP, Shank and Homer (Zeng et al., 2018). We speculate whether phase separation by the PSG:ADGRB1 complex is associated with PSD anchoring to the membrane bilayer. It is known that PSD-95 can anchor to the membrane bilayer by binding via a second supramodule of PSD-95, the PDZ1-2 tandem, to a PBM from the intracellular C-terminal part of NMDA (Zeng et al., 2018) or AMPA receptors (Matt et al., 2019). Our current data suggest the possibility of another anchoring point for PSD-95 with the membrane bilayer through the C-terminal part of ADGRB1.

LLPS is concentration-dependent; therefore, liquid droplets of PSG:FL CC ADGRB1 will appear only at a certain concentration threshold. Reduced ADGRB1 expression could decrease the level of ADGRB1 below the LLPS threshold for the PSG:ADGRB1 complex. Zeng et al. reported the first phase transition with PSG through interaction with SynGap (Zeng et al., 2016). They suggested that the dosage sensitivity of SynGap for synaptic functions is a general mechanism for synaptic proteins that undergo LLPS. Whereas both PSG and PDZ1-2 bind to ADGRB1, phase separation seems to be induced by the PSG supramodule, as suggested by its lower LLPS concentration threshold, which is below *K*_*d*_ for the PSG:ADGRB1 interaction, upon PSD reconstruction. Thus, multivalent interactions involving an extended interaction interface between PSG and the coiled-coil of ADGRB1 or SynGap are essential for LLPS, consistent with previous findings for protein:protein interactions to induce phase separation (Banjade and Rosen, 2014; Li et al., 2012; Zeng et al., 2016).

In summary, through our screen of proteins expressed in the PSD, we found that ADGRB1, like SynGap, binds specifically to the multidomain protein PSD-95. Furthermore, supertertiary structure and multivalent interactions alter affinity, specificity, and phase separation of PSD-95 interactions. Our data also suggest a putative role for ADGRB1 in the regulation of synaptic plasticity by anchoring PSD-95 to the postsynaptic membrane bilayer.

### Limitations of the study

Co-localization experiments in HeLa cells were performed by overexpression of GFP or mCherry-tagged proteins owing to limitations in the detection of endogenous proteins in cells and the unavailability of validated antibodies against the respective proteins. LLPS is observed and described in an artificial system *in vitro*, where we only use the C-terminal part of ADGRB1 or SynGap. The common experimental approaches to study LLPS, which are applied in this study, are limited to soluble proteins, therefore it is not possible with the chosen methods to study LLPS with FL ADGRB1 as it is a membrane-attached protein. Affinity measurements are also performed in *in vitro* systems. Whereas absolute affinities might be different *in vivo*, it is usually assumed that *differences* in affinity are similar *in vitro* and *in vivo* such that, for example, wild-type proteins and mutational variants can be compared and the results can be extrapolated to an *in vivo* situation.

## STAR★Methods

### Key resources table

**Table:** 

REAGENT or RESOURCE	SOURCE	IDENTIFIER
**Bacterial and virus strains**		

BL21(DE3)pLysS	Invitrogen	C606003

**Chemicals, peptides, and recombinant proteins**		

Tween 20	VWR	663684B
Dimethylformamide (DMF)	Biosolve	UN2265
Piperidine	Biosolve	UN2401
N,N′-diisopropylcarbodiimide (DIC)	Biosolve	UN2929
Trifluoroacetic acid (TFA)	Biosolve	UN2699
OxymPure	Iris Biotech	RL-1180
Diethylether	Sigma-Aldrich	296082
Trissopropylsilane (TIPS)	Sigma-Aldrich	233781
Fmoc-L-Ala-OH	Gyros Proteintechnologies	SMP-30-A
Fmoc-L-Arg(pbf)-OH	Gyros Proteintechnologies	SMP-30-RBF
Fmoc-L-Asn(Trt)-OH	Gyros Proteintechnologies	SMP-30-NT
Fmoc-L-Asp(tbu)-OH	Gyros Proteintechnologies	SMP-20-DB
Fmoc-L-Gly-OH	Gyros Proteintechnologies	SMP-30-G
Fmoc-L-Gln(Trt)-OH	Gyros Proteintechnologies	SMP-20-QT
Fmoc-L-Glu(tbu)-OH	Gyros Proteintechnologies	SMP-30-EB
Fmoc-L-His(Trt)-OH	Gyros Proteintechnologies	SMP-30-HT
Fmoc-L-Ile-OH	Gyros Proteintechnologies	SMP-30-I
Fmoc-L-Leu-OH	Gyros Proteintechnologies	SMP-30-L
Fmoc-L-Lys(Boc)-OH	Gyros Proteintechnologies	SMP-30-KBC
Fmoc-L-Met-OH	Gyros Proteintechnologies	SMP-30-M
Fmoc-L-Phe-OH	Gyros Proteintechnologies	SMP-30-F
Fmoc-L-Pro-OH	Gyros Proteintechnologies	SMP-30-P
Fmoc-L-Ser(tbu)-OH	Gyros Proteintechnologies	SMP-30-SB
Fmoc-L-Thr(tbu)-OH	Gyros Proteintechnologies	SMP-30-TB
Fmoc-L-Tyr(tbu)-OH	Gyros Proteintechnologies	SMP-30-YB
Fmoc-L-Trp(Boc)-OH	Gyros Proteintechnologies	SMP-30-WBC
Fmoc-L-Val-OH	Gyros Proteintechnologies	SMP-30-V
Fmoc-L-Ile-HMPA-Polystyrene Resin	Merck Millipore	856011
Fmoc-L-Leu-HMPA-Polystyrene Resin	Merck Millipore	856012
Fmoc-L-Phe-HMPA-Polystyrene Resin	Merck Millipore	856015
Fmoc-L-Ser(tbu)-HMPA-Polystyrene Resin	Merck Millipore	856016
Fmoc-L-Val-HMPA-Polystyrene Resin	Merck Millipore	856020
TCEP-HCL	Thermo Scientific	20491
Ni Sepharose® 6Fast Flow	Cytiva	GE17-5318-01
Disuccinimidyl glutarate (DSG)	ThermoFisher	A35392
DMEM	Gibco	61965026
ProLong Diamond Antifade Mountant	Life Technologies	P36965
Triton X-100	Sigma	X-100
Fetal bovine serum, heat-inactivated	Gibco	10500064
PenStrep (penicillin G and streptomycin sulfate)	Gibco	15140122
TurboFect	Thermo Scientific	R0531
DAPI	Sigma	D9542
FITC-PLVGQDIIDLQTEV	Genecust	FITC-14 AA ADGRB1
FITC-EERASSPAQCVTPVQTVV	Genecust	FITC-18 AA MTMR2
FITC- YKKVLDTKNYKQTSV	Genecust	FITC-15 AA CRIPT
YKKVLDTKNYKQTSV	Genecust	unlabeled 15 AA CRIPT
DNS-GKKVLDTKNYKQTSV	Genecust	15 AA CRIPT
DNS-AQRGSFPPWVQQTRV	Genecust	15 AA SynGap
DNS-RDGCHRLPKIKETTV	Genecust	15 AA FRPD4
DNS-AASNTNTLNRKTTPV	Genecust	15 AA CCG8
DNS-LTAAKPKRSFIESNV	Genecust	15 AA TANC1
DNS-APDSKRQSILFSTEV	Genecust	15 AA BCR
DNS-QNSTNLPHGHSTTRV	Genecust	15 AA NLGNX
DNS-KTESKKHGRWKLWFL	Genecust	15 AA INSY2
Ac-YKKVLDTKNYKQTSV	Novo Nordisk	CRIPT
Ac-YHPHPHPHSHSTTRV	Novo Nordisk	NLGN1
Ac-YDGSHRLPKIKETTV	Novo Nordisk	FRPD4
Ac-YSSTSPQSTDKSSTV	Novo Nordisk	SRGP2
Ac-YYETGSLSFAGDERI	Novo Nordisk	SEZ6
Ac-YEEELQDSRVYVSSL	Novo Nordisk	CAC1C
Ac-YKEGYNVYGIESVKI	Novo Nordisk	GRIA2
Ac-YMSHSSGMPLGATGL	Novo Nordisk	GRIA1
Ac-YDSTEQSDVRFSSAV	Novo Nordisk	GPER1
Ac-YGADIPYEDIIATEI	Novo Nordisk	LZTS1
Ac-YPEYSGGNIVRVSAL	Novo Nordisk	KCND2
Ac-YRRVYKKMPSIESDV	Novo Nordisk	NMDE1
Ac-YLADEVSASLAKQGL	Novo Nordisk	TAU
Ac-YVMQDESFPASKIEL	Novo Nordisk	MAP1B
Ac-YWTPSRLERIESTEI	Novo Nordisk	LZTS3
Ac-YHKGEVVDVMVIGRL	Novo Nordisk	GEPH
Ac-YDSIEIYIPEAQTRL	Novo Nordisk	DLGP2
Ac-YESIEIYIPEAQTRL	Novo Nordisk	DLGP1
Ac-YVRLLQSDPSSASQF	Novo Nordisk	HPCA
Ac-YEPPEPKKSRRSVLL	Novo Nordisk	CDC42
Ac-YRTQMQQMHGRMVPV	Novo Nordisk	EPHA4
Ac-YGQRDLYSGLNQRRI	Novo Nordisk	CD3E
Ac-YEPGTELSPTLPHQL	Novo Nordisk	KCC1A
Ac-YFTATEPQYQPGENL	Novo Nordisk	FYN
Ac-YFTYHTSDGDALLLL	Novo Nordisk	CX3C1
Ac-YYETSHYPASPDSWV	Novo Nordisk	CTND2
Ac-YLSGPYIWVPARERL	Novo Nordisk	DLG4
Ac-YEKAASREEPIEWEF	Novo Nordisk	CUX1
Ac-YDMRLQMNQTLPVQV	Novo Nordisk	EPHB3
Ac-YERAANREEALEWEF	Novo Nordisk	CUX2
Ac-YDKRDSEEESESTAL	Novo Nordisk	RHG44
Ac-YPQHLRTASKNEVTV	Novo Nordisk	SHSA7
Ac-YHHTSYTASKTEVTV	Novo Nordisk	SHSA6
Ac-YRQPLPDSNPEESSV	Novo Nordisk	SEM4C
Ac-YTAAKPKRSFIESNV	Novo Nordisk	TANC1
Ac-YLTGAPLATSDETSI	Novo Nordisk	SEM4F
Ac-YMEGLVPVGYTSLVL	Novo Nordisk	RUSC1
Ac-YLPLDVQEGDFQTEV	Novo Nordisk	AGRB3
Ac-YPLVGQDIIDLQTEV	Novo Nordisk	ADGRB1
Ac-YQETLFVGNDQVSEI	Novo Nordisk	TM108
Ac-YDKPLSKKHAHSVNF	Novo Nordisk	PDLI5
Ac-YNSTNLPHGHSTTRV	Novo Nordisk	NLGNX
Ac-YPSIVQALSLYDGLV	Novo Nordisk	NCS1
Ac-YFYYLYSMVYTLVSF	Novo Nordisk	CNIH2
Ac-YSLSKHESEYNTTRV	Novo Nordisk	NETO1
Ac-YQEQNKVLWIPASPL	Novo Nordisk	DLG5
Ac-YLHGHRYSSGSRSLV	Novo Nordisk	IQEC3
Ac-YTESKKHGRWKLWFL	Novo Nordisk	INSY2
Ac-YPDSKRQSILFSTEV	Novo Nordisk	BCR
Ac-YEPSERHTEEALRKF	Novo Nordisk	ANS1B
Ac-YVESLSSESTATSPV	Novo Nordisk	TNR16
Ac-YAPATSPPERALSKL	Novo Nordisk	TUTLB
Ac-YHGGNLETREPTNTL	Novo Nordisk	GRIP1
Ac-YQSGPFIWIPSKEKL	Novo Nordisk	DLG2
Ac-YQSGSYIWVPAKEKL	Novo Nordisk	DLG1
Ac-YDVLKNMTDKAPPGV	Novo Nordisk	CK2N1
Ac-YASNTNTLNRKTTPV	Novo Nordisk	CCG8
Ac-YAVALIAYLSKNNHL	GLB	SYNG1
Ac-YQARRGKKKSGSLVL	GLB	RHOA
Ac-YALRMRMAKLGKKVI	GLB	PALMD
Ac-YSRQHSKLLDFDDVL	GLB	NSMF
Ac-YLKSPDYDQMSSSPC	GLB	CCG5
Ac-YQRGSFPPWVQQTRV	GLB	SYNGAP⍺1
Ac-YERLKQFVSQELVNL	GLB	AB17B
Ac-YQPYFITNSKTEVTV	Novo Nordisk	SHSA9
Ac-YPIGGSLNSTTDSAL	Novo Nordisk	RGS14
Ac-YAELKRNTLYFSTDV	GLB	ABR
Ac-YLFASLEAYSHIRGF	Novo Nordisk	NAC2
Ac-YDVVAVYPNAKVELV	Novo Nordisk	PDLI4
Ac-YPALPTVSISSSAEV	Novo Nordisk	DOC10
Ac-YESTTEEIELEDVEC	GLB	VSTM5
Ac-YPPSSPTLSVSSTSL	GLB	KLH17
Ac-YLFATLEAYSYIKGF	GLB	NAC3
Ac-YSSPAQSVTPVQTVV	GLB	MTMR2

**Experimental models: Cell lines**		

HeLa (CCL-2)	American Type Culture Collection	n.a.

**Recombinant DNA**		

MG3C-SynGAP CC-PBM WT	(Zeng et al., 2016)	n.a.
32M3C-GKAP 3GBR-CT	(Zeng et al., 2018)	n.a.
M3C-Homer 3 EVH1-CC WT	(Zeng et al., 2018)	n.a.
M3C-Shank3 NPDZ-HBS-CBS-SAM M1718E	(Zeng et al., 2018)	n.a.
PSD-95 FL	(Nissen et al., 2015)	n.a.
PDZ3 (PSD-95)	(Gianni et al., 2005)	n.a.
PSG (PSD-95)	(Laursen et al., 2020a)	n.a.
PSG G322A (PSD-95)	(Laursen et al., 2020b)	n.a.
PSG G335A (PSD-95)	(Laursen et al., 2020b)	n.a.
PSG G322A G335A (PSD-95)	This paper	n.a.
PSG F325A (PSD-95)	(Laursen et al., 2020b)	n.a.
PSG I327V (PSD-95)	(Laursen et al., 2020b)	n.a.
PSG F325A I327V (PSD-95)	This paper	n.a.
MG3C-ADGRB1 CC-PBM WT	This paper	n.a.
PDZ1-2 (PSD-95)	(Chi et al., 2010)	n.a.
mCherry-PSG	This paper	n.a.
mCherry-PDZ1-2	This paper	n.a.
EGFP-FL CC SynGap	This paper	n.a.
EGFP-FL CC ADGRB1	This paper	n.a.
PreScission protease	n.a.	n.a.

**Software and algorithms**		

Prism 9.0	GraphPad	GraphPad Prism
ASTRA	Wyatt Technology	n.a.
Image Lab	BioRad	n.a.
Pro-Data Viewer	Applied Photophysics (stopped flow)	n.a.
Origin 7.0	Malvern instruments ITC software	n.a.

**Other**		

SX-17 MV stopped-flow spectrophotometer	Applied Photophysics	n.a.
iTC200 microcalorimeter	Malvern instruments	n.a.
SEC-MALS	Agilent HPLC system coupled to an in-line MALS system (Wyatt)	n.a.
Superdex 200, increase 10/300	Cytiva	17-5175-01
Nikon eclipse 90i microscope	Nikon	n.a.
4–20 % Mini-PROTEAN® TGX™ Precast Protein Gels 15 wells	BioRad	4561096
Nickel Sepharose Fast Flow column	GE Healthcare	n.a.
S-100	GE Healthcare	n.a.
MALDI-TOF mass spectrometry	n.a.	n.a.
Corning® 96 well NBS™ Microplate	Corning Life Sciences	CLS3650
96 Well 1.2mL Polypropylene Deepwell Storage Plate	Thermo Scientific	AB-1127
SpectraMax iD5	Molecular Devices	n.a.
NanoDrop™ One^C^	Thermo Scientific	840274200
JASCO J-1500	Jasco	n.a.
ZEISS Axio Observer Inverted Microscope	Zeiss	n.a.

### Resource availability

#### Lead contact

Any request for resources or constructs should be directed to Per Jemth (per.jemth@imbim.uu.se).

#### Materials availability

All unique constructs generated in this study are available from the lead contact.

### Method details

#### Identification of putative PDZ-binding proteins in the PSD, peptide library design and peptide synthesis

High coiled coil content and affinity are properties reported to be important for SynGap to undergo LLPS with PSD-95. Coiled coil percentage was calculated for all proteins in the library using online available software and compared with the *K*_i_ value reported from binding experiments (Figure 2). We picked MTMR2 and ADGRB1 for further analysis and expressed their FL CC domain, as they had a high coiled coil percentage and a decent affinity for PDZ3.

We searched manually in Uniprot for proteins predicted to be located in proximity to PSD-95 in PSD. In more detail, we used term-search in Uniprot using dendrite, post synaptic density, post synaptic density membrane and dendrite spine, respectively. It is well-known that a hydrophobic amino acid at the C-terminus (denoted P_0_) is essential for binding to PDZ3 in PSD-95. Thus, 74 out of 80 C-terminal peptides from proteins with Leu, Ile, Cys, Phe or Val at their C-terminal position (denoted P_0_ according to established PDZ nomenclature) were included in the final peptide library. We limited the study to the main isoform of proteins in proximity to PSD-95 to reduce the size of the peptide library, and splice variants as well as peptides that proved difficult to handle were therefore excluded. Peptides were designed with a free C-terminal, 14 native amino acids, a Tyr at P_-14_ for concentration determination by absorbance, and with acetylated N-terminal.

All peptides for fluorescence polarization experiments were synthesized by parallel 96 format peptide synthesis using Intavis multipep RSi. 10 mg per well of Fmoc-preloaded resins were used. Fmoc-amino acids (Fmoc-AA) 0.3 M in dimethylformamide (DMF) containing 0.3 M OxymaPure were used. Three couplings using 100 μL Fmoc-AA and 10 μL DIC (3 M in DMF) were employed with coupling times of 5, 15 and 60 min. Removal of Fmoc was performed by washing twice with 120 μL of 25% piperidin in DMF for 2 and 8 min. Trifluoroacetic acid (TFA) cleavage was done by 92% TFA containing 4% triisopropylsilane, 1% thioanisol, and 3% H_2_O for 2 hours. A total volume of 1 mL TFA was added to each well. TFA was reduced in volume to approximately 150 μL by N_2_ flow followed by precipitation by the addition of diethylether. The peptides were transferred to Waters solvinert plates and washed thoroughly five times with diethylether. After washing, the peptides were dried and then dissolved in neat DMSO. Peptides were analyzed by UPLC-MS and showed an average purity of 85%.

Several of the peptides were re-ordered from GL Biochem (see Data S1). 15 AA peptides for stopped flow analysis with dansylated N-terminal and 15 AA CRIPT, 14 AA ADGRB1 and 18 AA MTMR2 with FITC labelled N-terminal for fluorescence polarization experiments were ordered from Genecust.

#### Protein expression and purification

PDZ3, PSG, PSG_G322A_, PSG_F325A_, PSG_I327V_, PSG_G335A_, PSG_G322A G335A_, PSG_F325A I327V_ and PDZ1-2 were expressed and purified as previously described (Laursen et al., 2020b). Plasmids (pETMG3C) encoding GB1-His_6_ tag full length coiled coil (FL CC) SynGap, MTMR2 or ADGRB1 were transformed into *Escherichia coli* BL21(DE3) pLys cells (Invitrogen). Cells were first grown in LB medium at 37°C in a rotary shaker. Overexpression of proteins was induced with 1 mM isopropyl-β-D-1-thiogalactopyranoside at OD_600_ of 0.6–0.8, and the cells were incubated 4 hours at 30°C (FL CC ADGRB1 and FL CC MTMR2) or overnight at 18°C for FL CC SynGap. Proteins were purified from the soluble fraction on a Nickel Sepharose Fast Flow column (GE Healthcare) equilibrated with 50 mM Tris pH 7.8, 200 mM NaCl, 10% glycerol, 20 mM Imidazole and 0.5 mM DTT. Bound proteins were eluted by increasing the imidazole concentration to 250 mM. Proteins were dialyzed into 50 mM Tris pH 7.8, 200 mM NaCl, 10% glycerol and 1 mM DTT and tag-cleaved by precision enzyme overnight. Cleaved proteins were loaded onto Nickel Sepharose Fast Flow columns, and the unbound flow through fraction was collected, concentrated and further purified using size exclusion chromatography (S-100, GE Healthcare). Protein purity was quantified by SDS-PAGE and identity by MALDI-TOF mass spectrometry. PSD-95, GKAP, Shank and Homer were expressed and purified in a similar fashion as SynGap. These proteins were characterized in previous studies (Christensen et al., 2022; Zeng et al., 2018).

#### Fluorescence polarization assay

The affinity of the probe in the FP experiments, FITC-15 AA CRIPT, was determined using a saturation binding experiment for each of the protein variants as previously described (Bach et al., 2012). In short, increasing concentration of protein was added to a fixed (5 nM) concentration of probe. Binding experiments were performed in 50 mM sodium phosphate pH 7.45, 21 mM KCl (total ionic strength, I = 150 mM), 0.1% Tween 20 and 1 mM Tris-2-carboxyethyl-phosphine (TCEP) at room temperature in black, non-binding surface, flat bottom 96 well plates (Corning Life Sciences). Samples were measured in a plate reader (SpectraMax iD5, Molecular Devices) at room temperature and at excitation/emission wavelengths of 485/535 nm. The fluorescence polarization (FP) value from the probe in absence of protein (background signal) was subtracted from all raw FP values to obtain specific fluorescence polarization values. The specific FP value was plotted as a function of increasing protein concentration (two-fold concentration difference between each data point) and data were fitted to a hyperbolic function accounting for a 1:1 binding in GraphPad Prism 9.0.

(Equation 1) $$\text{Y}={\text{B}}_{\max}*\left[\text{protein}\right]/\left(K_{d}+\left[\text{protein}\right]\right)$$

*K*_*d*_ is the equilibrium dissociation constant, B_max_ is the maximum amplitude of fluorescence polarization and Y is the fluorescence polarization value.

To determine the affinity of peptides from the C-terminal library we used non-labeled peptides with acetylated N-termini in a competition assay. The concentration of protein was adjusted, based on the *K*_d_ value from the saturation experiment, to obtain 50–80% of B_max_ as the starting FP signal: PDZ3 (1.3–6 μM), PSG (2–3 μM), PSG_G322A_ (0.5 μM), PSG_G335A_ (0.6 μM), PSG_G322A G335A_ (0.3 μM), PSG_F325A_ (3.8–7 μM), PSG_I327V_ (6–12 μM) and PSG_F325A I327V_ (10–14 μM). PDZ3 and each PSG variant were incubated with a fixed concentration of FITC-15 AA CRIPT (5 nM). FITC-15 AA CRIPT was displaced upon addition of non-labeled peptide in excess (two-fold difference in concentration between each data point). Conditions and experimental setup were as described for the saturation experiment. Fluorescence polarization values as a function of the logarithmic value of non-labeled peptide concentration were fitted to a sigmoidal dose-response equation:

(Equation 2) $$\text{Y}={\text{B}}_{\text{Bottom}}+\left({\text{B}}_{\text{Top}}-{\text{B}}_{\text{Bottom}}\right)/\left(1+10ˆ\left(\text{Log}\left({\text{IC}}_{50}-\left[\text{Peptide}\right]\right)*\text{n}\right)\right)$$

Y is the fluorescence polarization value, B_Bottom_ is the fluorescence polarization signal of probe in absence of protein, (B_Top_-B_Bottom_) is the amplitude of fluorescence polarization signal, IC_50_ is concentration of peptide giving 50% inhibition, i.e., when the fluorescence polarization value is at the midpoint of the transition, [Peptide] is concentration of non-labeled peptide, and n is the Hill slope. The IC_50_ values were converted to *K*_i_ as previously described (Nikolovska-Coleska et al., 2004) (Figure 1D). All saturation experiments were performed in three replicates of technical triplicates, whereas competition experiments were performed in technical triplicates.

#### Circular dichroism measurements

Far-UV spectra of proteins were acquired on a JASCO J-1500 spectrapolarimeter from 260 to 200 nm (average of 5 scans) to check that the proteins were folded. Proteins (20 μM) were diluted in 100 mM NaCl, 50 mM Tris pH 7.8 and 1 mM TCEP at 25 °C.

#### Liquid liquid phase separation assays

Liquid droplet phase transition was assayed directly by light microscopy, sedimentation or turbidity. LLPS appeared by mixing PSG, PSG_G322A_, PSG_F325A_, PSG_I327V_, PSG_G335A_, PSG_G322A G335A_ or PSG_F325A I327V_ with FL CC SynGap or FL CC ADGRB1. All proteins were prepared in 100 mM NaCl, 1mM TCEP and 50 mM Tris, pH 7.8.

Liquid phase droplets were imaged by phase contrast microscope Zeiss Axio Observer Inverted Microscope by mixing of PSG and FL CC SynGap or FL CC ADGRB1 in a 1:1 or 1:3 stoichiometry at final concentrations of 50:50 μM (PSG: FL CC SynGap), 50:50 μM (PSG_G322A G335A_:FL CC SynGap), 34:102 μM (PSG_G322A G335A_:FL CC ADGRB1) and 42.5:128 μM (PSG:FL CC ADGRB1) on a glass slide.

The solution turbidity assay was performed by challenging PSG (15 μM), PSG_G322A G335A_ (15 or 28 μM) or PDZ1-2 (40 μM) with increasing concentrations of FL CC SynGap (15 to 70 μM) or FL CC ADGRB1 (50 to 206 μM) and measuring absorbance at 350 nm over 10 min on a JASCO J-1500 spectrapolarimeter at 25°C. As expected, the traces showed a complex behavior including a lag phase, reminiscent of protein aggregation data. Experimental traces were analyzed to obtain the maximum growth rate expressed as absorbance units per minute. Traces for high affinity PSG variants that did not display a lag phase were fitted to a double exponential function. The maximum rate in these cases is the initial rate, i.e., the slope of the curve at time zero. In experiments with a lag phase we used the following equation (Buchanan and Cygnarowicz, 1990):

C+A∗exp(−exp(−B(t−M)))

where M is the time point of maximum growth rate, t is the time and B is the relative growth rate at time M. A and C are constants that account for the amplitude of the trace and the offset. The maximum rate was calculated from the first derivate of the equation at time M.

The same samples used for sedimentation assay were incubated overnight at 4°C, followed by centrifugation for 20 min at max speed in a table top microtube centrifuge at room temperature. Supernatant and pellet were separated. The pellet was resuspended in a volume equal to that of the supernatant. Proteins were analyzed by SDS-PAGE gradient gel (4–20%) with Coomassie blue staining. Band intensities were quantified by Image Lab software from BioRad.

#### Reconstituted PSD by phase transition sedimentation assay

PSD-95, Homer, Shank, GKAP, FL CC SynGap, FL CC ADGRB1, PDZ1-2 and PSG were prepared in 50 mM Tris, 100 mM NaCl and 2 mM TCEP, final concentration of 20 μM (except FL CC ADGRB1 0–100 μM) in a total volume of 70 μL with four to seven of the different proteins. GKAP, Shank, Homer and 0 to 100 μM ADGRB1 were present in all experiments. In separate experiments additional proteins were included: (1) PSD-95, (2) PSD-95 and SynGap, (3) PSG, PDZ1-2 and SynGap and (4) PSG and PDZ1-2. Samples were incubated for 10 min at room temperature and characterized as described above by SDS-PAGE gradient gel (4–20%) with Coomassie blue stain. Significance was evaluated by two-way ANOVA with Tukey test using Prism 9.0 software (GraphPad).

#### Chemical cross-linking assay

PSG and 15 AA peptides derived from 13 proteins (ADGRB1, SynGap, CRIPT, FRPD4, TANC1, NLGNX, BCR, CCG8, VSTM5, INSY2, CNIH2, SRCN1 and DRD2) were prepared in 50 mM sodium phosphate pH 7.45, 21 mM KCl (I = 150), 1 mM TCEP. Disuccinimidylglutarate (DSG) powder was dissolved in DMSO. DSG is an N-hydroxysuccinimide ester that reacts with the N-terminal primary amine of proteins or peptides, resulting in crosslinking. 15 AA SynGap binds to PSG and promotes association of the PSGs resulting in DSG-mediated crosslinking. The molar ratio of DSG to PSG was 10:1 and for PSG to 15 AA peptide the ratio was 1:2. PSG, DSG and peptide were mixed at room temperature and incubated for 30 min to allow N-terminal crosslinking of two PSG molecules. Cross linking reactions were quenched by incubating the samples for 30 min after addition of Tris pH 7.8 (final concentration 200 mM). PSG cross linking was analyzed by SDS-PAGE with Coomassie blue staining and quantified by Image lab software (Bio-rad).

#### Kinetic experiments

All kinetic experiments were performed in 50 mM sodium phosphate, pH 7.45, 21 mM KCl (I = 150) and 1 mM Tris-2-carboxyethyl-phosphine (TCEP) at 10°C. Binding and dissociation experiments were performed using an upgraded SX-17 MV stopped-flow spectrophotometer (Applied Photophysics, Leatherhead, UK) and carried out as described previously (Chi et al., 2010; Laursen et al., 2020b). Association rate constants (*k*_on_) were obtained from binding experiments under conditions approaching pseudo first order conditions at high ligand concentration using N-terminally dansylated peptides as ligand. Thus, PSG (1 μM final concentration) was mixed rapidly with increasing concentrations of dansylated ligand (final concentrations of 2 to 20 μM of 15 AA C-terminal peptide) derived from the following proteins: CRIPT, SynGap, FRPD4, TANC1, NLGNX, BCR, CCG8 and INSY2. The change in fluorescence emission upon PSG: 15 AA ligand interaction was measured with a 330 nm interference filter (330 ± 25 nm) using an excitation wavelength at 280 nm. Kinetic traces (at least 5 for each observed rate constant) were recorded, averaged, and fitted to a single exponential function (Laursen et al., 2020a) consistent with a two-state bimolecular association/dissociation mechanism. 15 AA SynGap displayed a more complex binding mechanism and data were fitted to a double exponential function. The fitting yielded observed rate constants (*k*_*obs*_) for each ligand concentration. A plot with *k*_*obs*_ versus ligand concentration was obtained and fitted to an equation derived for a second order bimolecular association reaction to account for the deviation from linearity at low CRIPT concentrations (Laursen et al., 2020b). The dissociation rate constant (*k*_*off*_) was obtained from displacement experiments when *k*_*off*_ < 20 s^−1^. PSG (2 μM) was mixed with dansylated 15 AA ligand (10 μM) followed by a long incubation (>15 min) to ensure that equilibrium was established. Dansylated 15 AA ligand was displaced from PSG by mixing with an excess of unlabeled 15 AA CRIPT (100, 150, 200 μM). *k*_*off*_ values were estimated from the average of three *k*_*obs*_ determinations at high concentration of unlabeled 15 AA CRIPT peptide, in a range where the *k*_*obs*_ values were constant with unlabeled 15 AA CRIPT concentration.

#### Isothermal titration calorimetry

ITC measurements were carried out on iTC200 microcalorimeter (Malvern instruments) at 25°C. Proteins were dialyzed in 50 mM Tris pH 7.8, 1 mM TCEP and 100 mM NaCl. PSG, PSG_G322A_, PSG_F325A_, PSG_I327V_, PSG_G335A_, PSG_G322A G335A_, PSG_F325A I327V_ or PDZ1-2 was loaded into the cell and FL CC ADGRB1 or 15 AA SynGap was loaded into the syringe. FL CC ADGRB1 or 15 AA SynGap was titrated (2 μL) 18 times into the cell with time intervals of 180 s. ITC titration data were analyzed by Origin 7.0 software and fitted to a one binding site model.

#### Size exclusion chromatography multi angle light scattering

SEC-MALS was done using Agilent HPLC system coupled to an in-line MALS system (Wyatt). Proteins were spun down or filtered before application to Superdex 200, increase 10/300 column equilibrated in 100 mM NaCl, 50 mM Tris pH 7.8, 1mM TCEP. Absorbance, refractive index and light scattering data was collected. Two runs were carried out for each protein (PSG, PDZ1-2 and PSG_G322A G335A_) alone and together with FL CC ADGRB1, which were mixed and incubated before application to Superdex 200. Data were analyzed using ASTRA software package and plotted using Prism 9.0 software (GraphPad).

#### Immunofluorescence microscopy

Immunofluorescence microscopy was performed as described previously (Inturi and Jemth, 2021). In brief, HeLa (CCL-2) cell lines obtained from American Type Culture Collection were cultured in DMEM supplemented with 10% (v/v) heat-inactivated fetal bovine serum and 1% (v/v) penicillin G and streptomycin sulfate solution (Gibco) at 37°C, 5% CO_2_. HeLa cells grown on 24-well cluster plates were co-transfected with 0.5 μg of mCherry-PSG or mCherry-PDZ1-2 plasmids with 0.5 μg of EGFP-FL CC SynGap or EGFP-FL CC ADGRB1 using turbofect transfection reagent (Thermo Scientific) as per the manufacturer’s instructions. Cells transfected only with 0.5 μg of mCherry-PSG or mCherry-PDZ1-2 or EGFP-FL CC SynGap or EGFP-FL CC ADGRB1 plasmids were added with 0.5 μg of empty vector plasmid to make a total of 1 μg per well. 24 hours post transfection, cells were fixed in 4% paraformaldehyde and permeabilized with 0.1% Triton X-100 for 15 min. Cells were washed three times with 2% BSA in PBS supplemented with 0.1% Tween 20 (PBST) and blocked with BSA/PBST for 30 min at room temperature. The DNA was labeled by incubation of cells with DAPI solution for 3 min, and washed three times with PBS. The cover slips were then mounted on slides with ProLong Diamond Antifade Mountant (Life Technologies) and imaged using a Nikon eclipse 90i microscope (Nikon).

### Quantification and statistical analysis

All data were analyzed using Prism 9.0 software (GraphPad).

Statistics was done using two-way ANOVA with Tukey test, ∗p < 0.05, ∗∗p < 0.01, ∗∗∗p < 0.001.

## Data Availability

All data reported in this paper will be shared by the lead contact upon request. This paper does not report an original code.

## Supplemental information

Document S1. Figures S1–S8

Data S1. Peptide sequences, and IC50 and *K*_*i*_ values from fluorescence polarization experiments, related to Figure 2 The excel file also includes peptide modifications, where they were synthesized and approximate purity.

Data S2. Data from stopped-flow kinetic experiments, related to Figure 3 The excel file also includes ITC and FP data that complement the kinetic data.
