# Supplementary material for: Determinants of affinity, specificity, and phase separation in a supramodule from Post-synaptic density protein 95
Source: iScience. 2022 Sep 5;25(10):105069. doi: 10.1016/j.isci.2022.105069 (PMC9490041; doi:10.1016/j.isci.2022.105069)
Supplement: Document S1. Figures S1–S8 [file mmc1.pdf]

iScience, Volume *25*

Supplemental information

Determinants of affinity, specificity, and phase separation in a supramodule from Post-synaptic density protein 95

Louise Laursen, Raviteja Inturi, Søren Østergaard, and Per Jemth

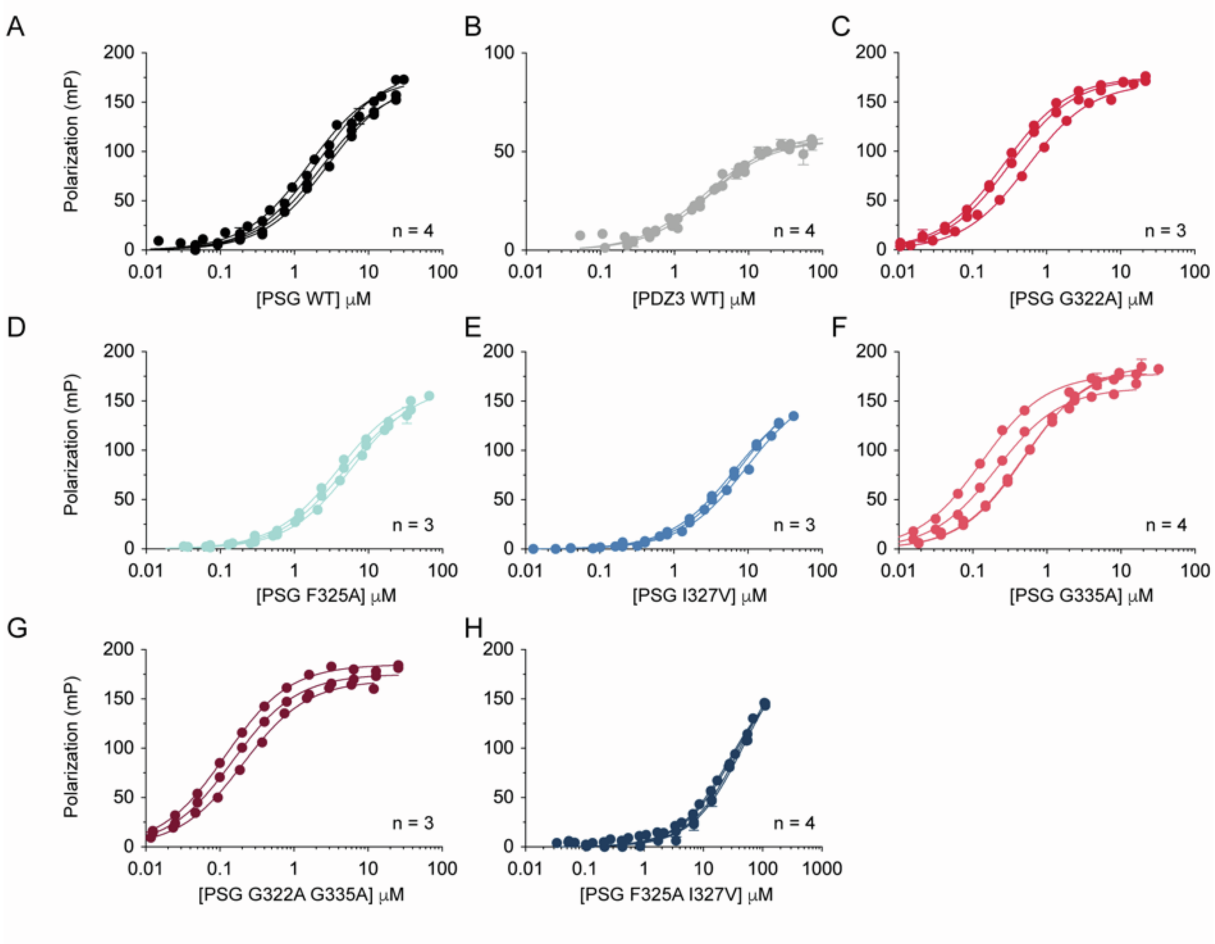

Figure S1. Affinity of the FITC-labeled 15 AA CRIPT probe for PSG variants and PDZ3. Related to Figure 1C and Supplementary Excel file 1.

Fluorescence polarization-monitored binding experiments of FITC-15 AA CRIPT to PDZ3 and the seven PSG variants included in the study: (A) PSG WT, (B) PDZ3, (C) PSG$_{G322A}$, (D) PSG$_{F325A}$, (E) PSG$_{I327V}$, (F) PSG$_{G335A}$, (G) PSG$_{G322A\ G335A}$ and (H) PSG$_{F325A\ I327V}$. Data were acquired at least in biological triplicates at room temperature in 50 mM Tris, 100 mM NaCl, 1 mM TCEP and 0.1 % Tween20. K_d values are shown in Fig. 1C.

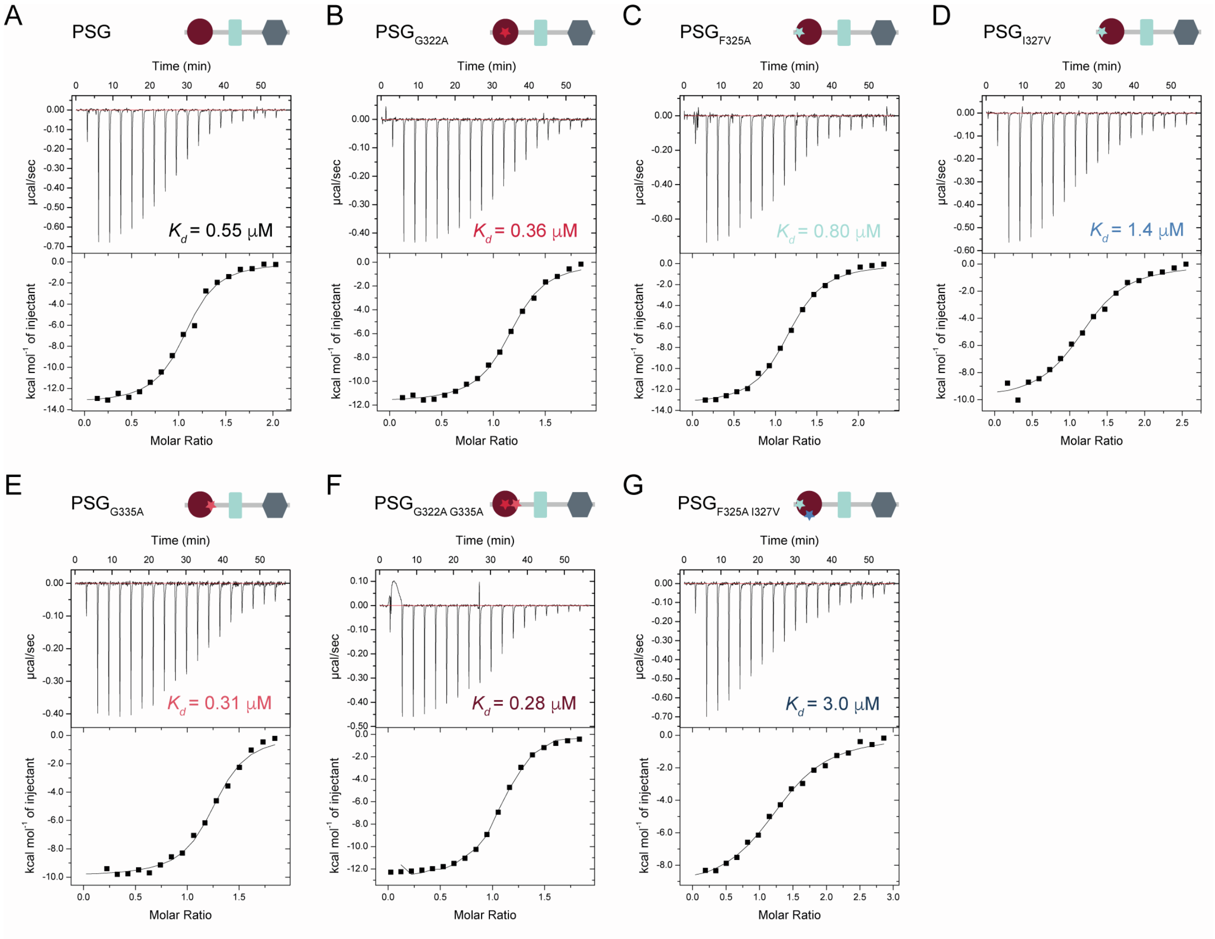

Figure S2: Isothermal titration calorimetry for SynGap 15 AA. Related to Figure 3.

ITC data for titration of SynGap 15 AA into (A) PSG, (B) PSG_{G322A}, (C) PSG_{F325A}, (D) PSG_{I327V}, (E) PSG_{G335A}, (F) $PSG_{G322A\ G335A}$ and (G) $PSG_{F325A\ I327V}$. Samples were dialyzed overnight in 50 mM Tris, 100 mM NaCl and 1 mM TCEP at 4°C and experiments were performed at 25°C.

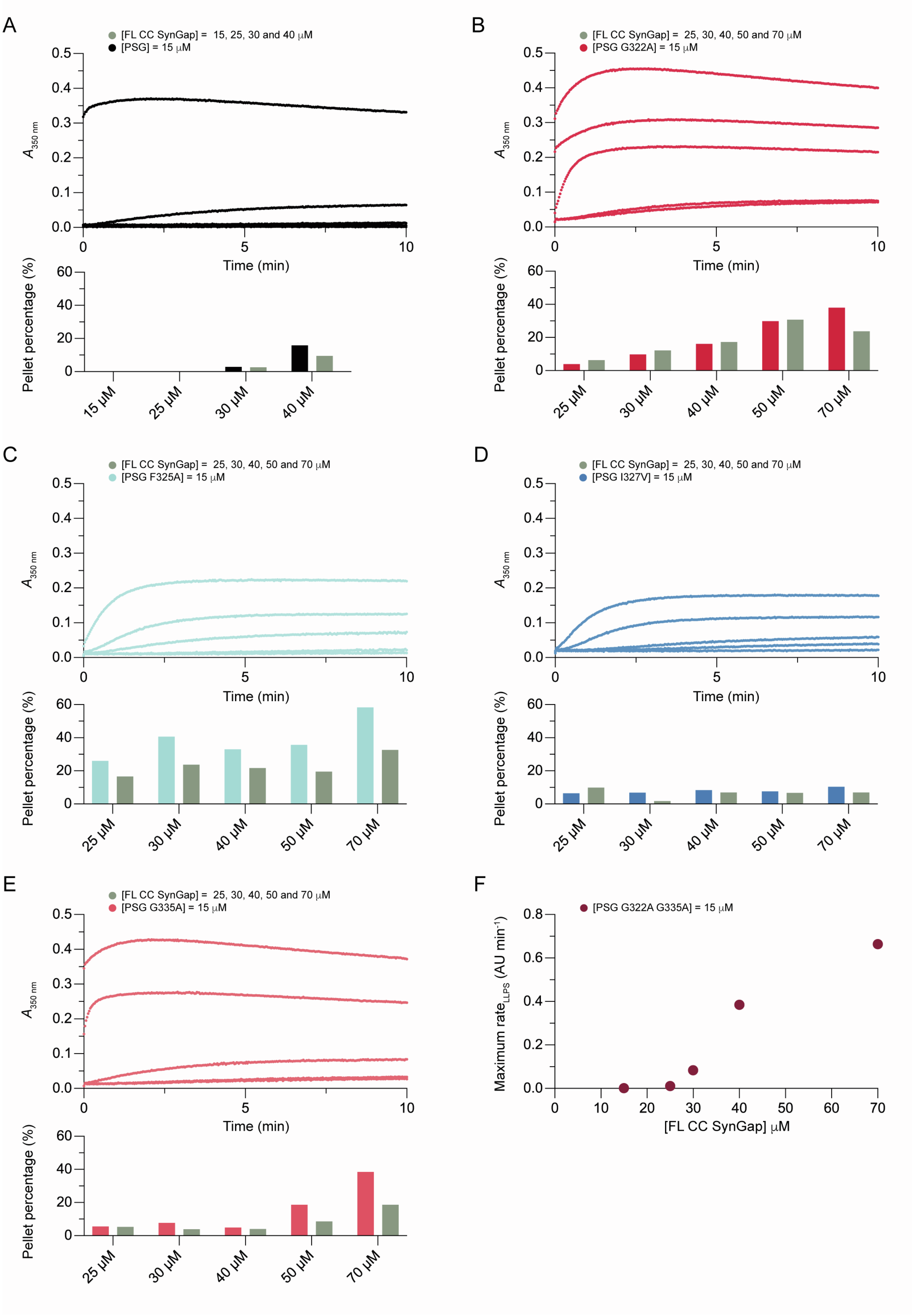
A
[FL CC SynGap] = 15, 25, 30 and 40 μM
[PSG] = 15 μM
$A_{350\ nm}$
Time (min)
Pellet percentage (%)
15 μM
25 μM
30 μM
40 μM
B
[FL CC SynGap] = 25, 30, 40, 50 and 70 μM
[PSG G322A] = 15 μM
C
[FL CC SynGap] = 25, 30, 40, 50 and 70 μM
[PSG F325A] = 15 μM
D
[FL CC SynGap] = 25, 30, 40, 50 and 70 μM
[PSG I327V] = 15 μM
E
[FL CC SynGap] = 25, 30, 40, 50 and 70 μM
[PSG G335A] = 15 μM
F
[PSG G322A G335A] = 15 μM
Maximum rate$_{LLPS}$ (AU min^{-1})
[FL CC SynGap] μM

Figure S3: Quantification of LLPS for all PSG variants. Related to Figure 4.

The top graphs in each panel illustrate LLPS formation over time as measured by light scattering at 350 nm at room temperature. The PSG variant concentration was held constant, and LLPS was monitored at different concentrations of FL CC SynGap. The rate of LLPS formation correlates with increasing FL CC SynGap concentration. Bottom graphs, LLPS was quantified by a sedimentation assay to determine the amount of FL CC SynGap and PSG in supernatant and condensed liquid phase (pellet). The bar graphs show the relative amount of LLPS (pellet fraction) as determined by SDS-PAGE of FL CC SynGap and PSG at the different concentrations of FL CC SynGap. (A) PSG, (B) PSG_{G322A}, (C) PSG_{F325A}, (D) PSG_{I327V}, and (E) PSG_{G335A}. (F) The observed maximum rate of LLPS growth as a function of FL CC SynGap concentration. Color code: black (PSG WT), red (PSG_{G322A}), light blue (PSG_{F325A}), blue (PSG_{I327V}), pink-orange (PSG_{G335A}), bordeaux ($PSG_{G322A\ G335A}$) and light green (FL CC SynGap). Data for $PSG_{F325A\ I327V}$ is not shown since no LLPS was observed in the concentration range 15 to 200 μM of FL CC SynGap.

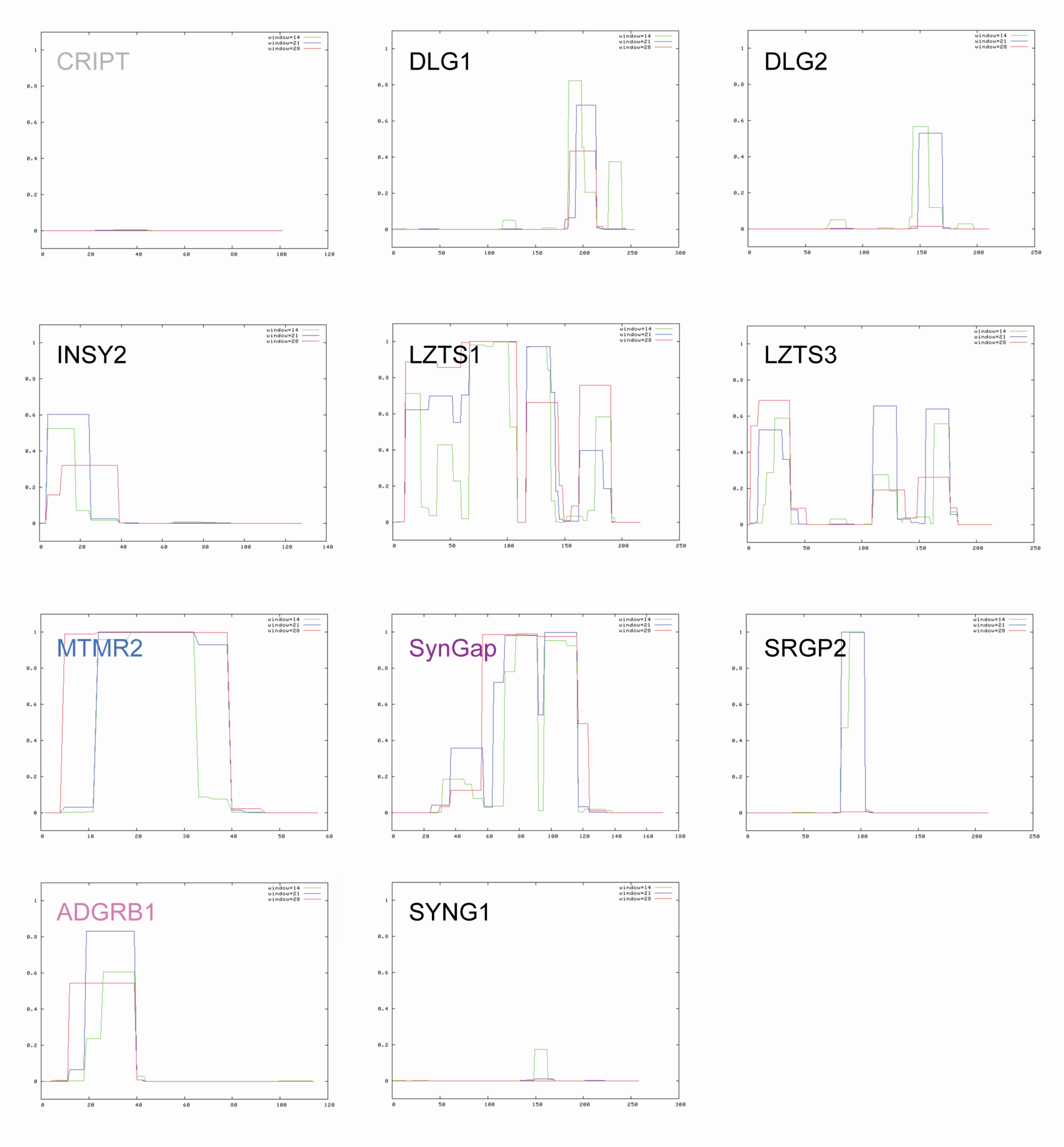

Figure S4: Coiled coil content of C-terminal regions of proteins from the PSD. Related to Figure 4 and Figure 5.

Nine proteins present in the PSD with high and two with low predicted coiled coil content. The coiled coil contents were predicted by the COILS software. CRIPT, MTMR2, SynGap and ADGRB1 were tested in this or in previous studies for LLPS formation with PSD-95.

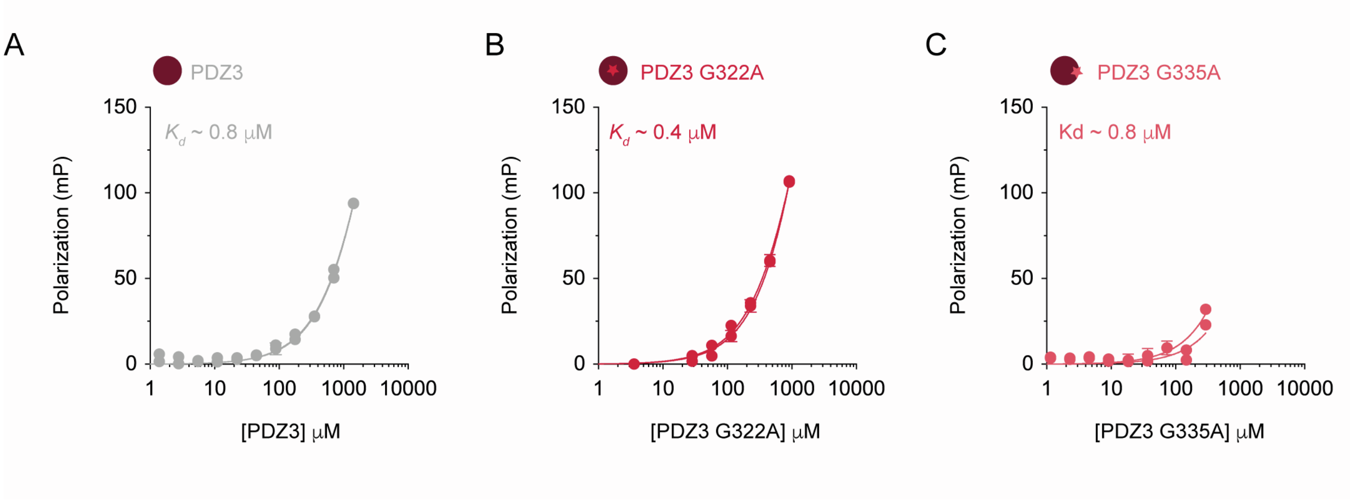

Figure S5: Weak binding of ADGRB1 peptide to PDZ3 variants. Related to Figure 5.
Fluorescence polarization-monitored binding experiments of FITC-labeled ADGRB1 14 AA to (A) PDZ3, (B) PDZ3$_{G322A}$, and (C) PDZ3$_{G335A}$. The experiments were performed in duplicate at room temperature in 50 mM Tris, 100 mM NaCl, 1 mM TCEP and 0.1 % Tween20.

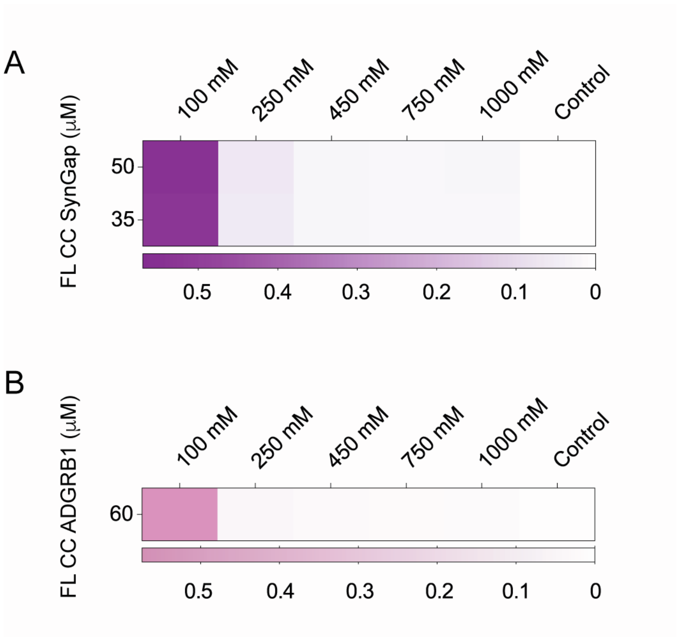

Figure S6: Salt dependence of phase separation. Related to Figure 5.
Salt dependence of phase separation was measured by a turbidity assay at 350 nm for the complex between PSG$_{G322A\ G335A}$ and (A) FL CC SynGap or (B) FL CC ADGRB1. Experiments were performed at 25°C in 50 mM Tris, 100 to 1000 mM NaCl and 1 mM TCEP. Color code: purple (max turbidity with FL CC SynGap) and pink (max turbidity with FL CC ADGRB1).

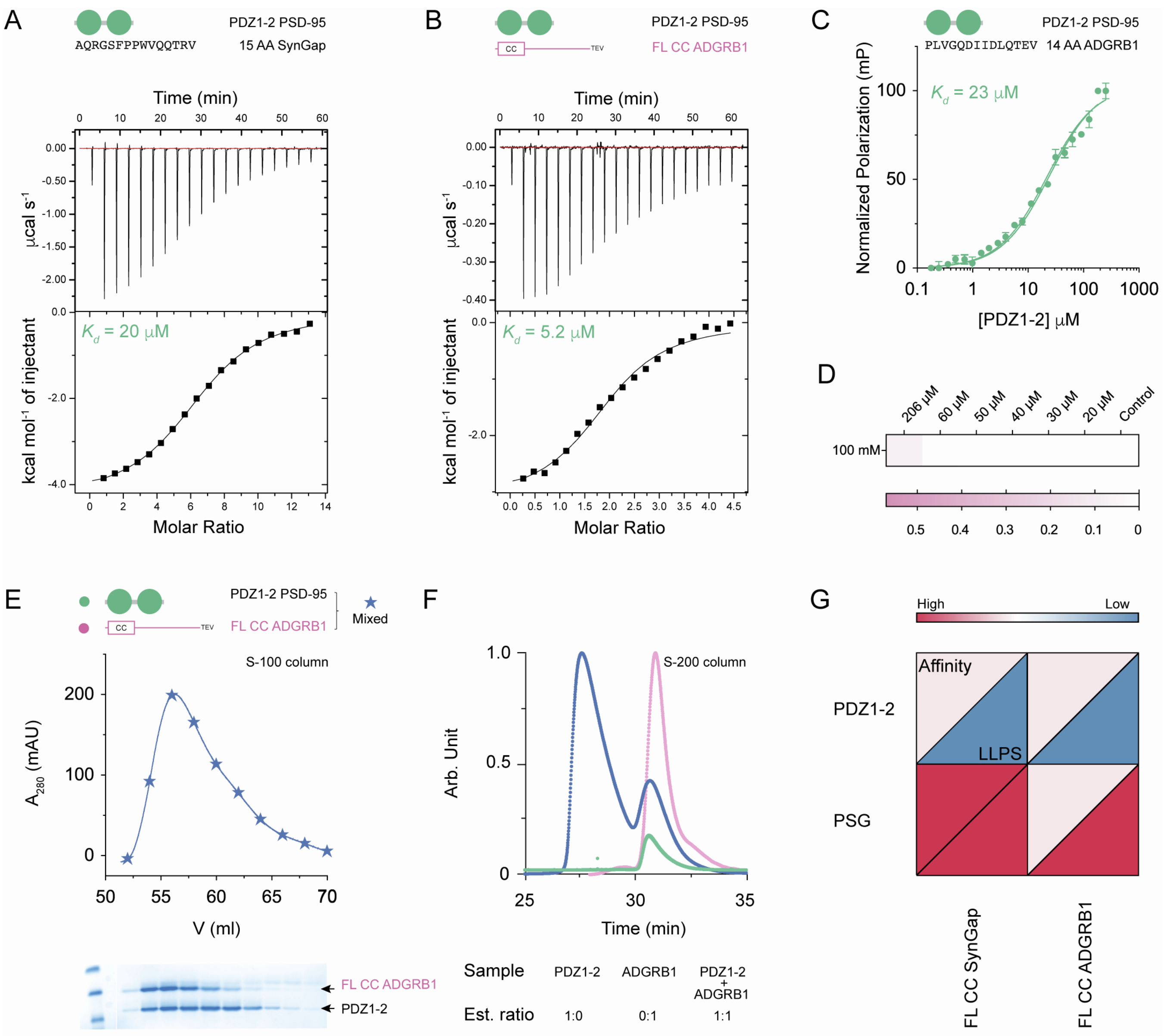

Figure S7: Affinity, specificity and phase separation threshold for PDZ1-2. Related to Figure 5.
ITC experiments to assess binding of (A) 15 AA SynGap and (B) FL CC ADGRB1 to PDZ1-2. (C) Binding between PDZ1-2 and 14 AA ADGRB1 measured by fluorescence polarization. (D) LLPS threshold measured for PDZ1-2 and FL CC ADGRB1 by a turbidity assay at 350 nm with a range of FL CC ADGRB1 concentrations (20 to 206 µM) in buffer with low salt for maximal LLPS conditions (100 mM NaCl, 50 mM Tris, 1 mM TCEP at 25°C). (E) Size exclusion chromatography elution profile of PDZ1-2 and FL CC ADGRB1 mixed in 1:1 ratio and SDS-PAGE showing the distribution of PDZ1-2 and FL CC ADGRB1 in each fraction. (F) SEC-MALS elution profiles of PDZ1-2, FL CC ADGRB1 and a PDZ1-2:FL CC ADGRB1 complex (blue). The elution profile of the complex shifted to the left and data fitting indicated a 1:1 complex between PDZ1-2 and FL CC ADGRB1. (G) Heatmap comparing the LLPS capacity and the binding affinity of FL CC SynGap and FL CC ADGRB1 to PDZ1-2 and PSG.

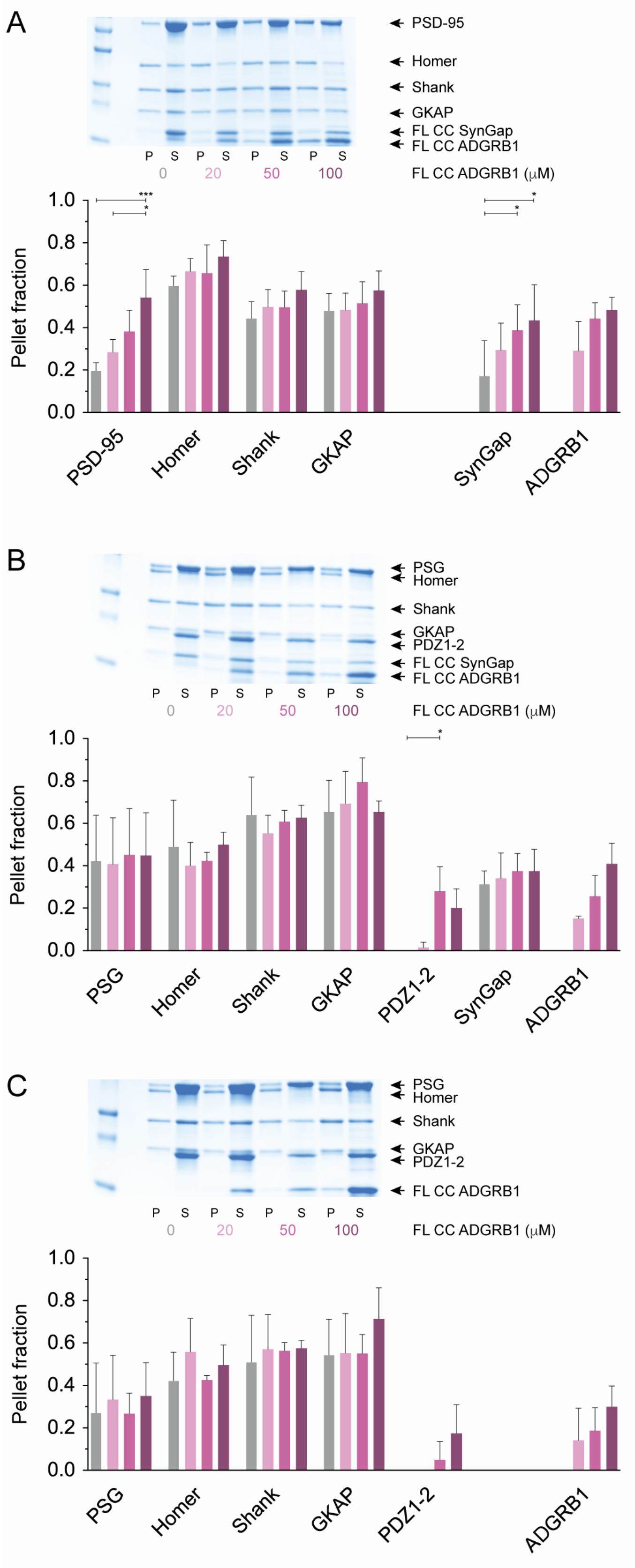

Figure S8: Reconstituted postsynaptic density (PSD). Related to Figure 5 and Figure S7. SDS-PAGE and quantification of the bands. Data show the distribution of PSD components in pellet (P) and supernatant (S) fractions in four different conditions: 0, 20, 50, and 100 mM FL CC ADGRB1 obtained from sedimentation assay. Thus, P represents the fraction forming LLPS. (A) The fractions of PSD-95 and SynGap in P increase significantly with increasing concentration of FL CC ADGRB1. (B) and (C) Comparison of the LLPS formation for PDZ1-2 and PSG with PSD components. (B) PDZ1-2 is dependent

of FL CC ADGRB1 to undergo LLPS, whereas PSG is independent, thus PSG can undergo LLPS with other PSD components. (C) Absence of FL CC SynGap reduces the overall amount of PSD components in the pellet fraction. A constant concentration of each PSD component was used (20 μM), except FL CC ADGRB1, which was as indicated in the figure. SynGap was absent in (C). Significance *, $p<0.05$; **, $p<0.01$; ***, $p<0.001$ was evaluated by two-way ANOVA with Tukey test using Prism 9.0 software (GraphPad).
